# Variable protein homeostasis in housekeeping and non-housekeeping pathways under mycotoxins stress

**DOI:** 10.1038/s41598-019-44305-0

**Published:** 2019-05-24

**Authors:** Yu Sun, Jikai Wen, Ruohong Chen, Yiqun Deng

**Affiliations:** 1 Guangdong Provincial Key Laboratory of Protein Function and Regulation in Agricultural Organisms, College of Life Sciences, South China Agricultural University, Guangzhou, Guangdong 510642 P.R. China; 2 Key Laboratory of Zoonosis of Ministry of Agriculture and Rural Affairs, South China Agricultural University, Guangzhou, Guangdong 510642 P.R. China

**Keywords:** Proteome informatics, Data integration

## Abstract

Transcript levels are the primary factor determining protein levels, but for the majority of genes, fold changes in transcript levels are larger than the corresponding changes in protein levels, a phenomenon that is termed “protein homeostasis”. However, this phenomenon is not well characterized in the context of environmental changes. In this study, we sequenced the entire transcriptome and proteome of chicken primary hepatocytes administered three mycotoxin treatments Aflatoxin B_1_ (AFB_1_), Ochoratoxin A (OTA) and Zearalenone (ZEN). Each mycotoxin induced unique set of differential expressed transcripts and proteins, suggesting variable cytotoxicity and biochemical action in cell. We found a weak positive correlation between transcript and protein changes, and the transcript changes were higher than the protein changes. Furthermore, we observed pathway-specific protein homeostasis pattern under mycotoxin stress. Specifically, the “Metabolism”, “Transcription” and “Translation” pathways (housekeeping pathways) showed lower fold changes in protein/mRNA levels than non-housekeeping pathways such as “Cell growth and death” and “Immune system”. Protein molecular weight had a weak negative effect on protein production, and this effect was stronger for non-housekeeping pathways. Overall, we hypothesize housekeeping pathways maintain stable protein production for baseline cellular function, whereas non-housekeeping pathways is associated with the fitness response to environmental stress.

## Introduction

The central dogma that the information flow in a biological system is generally from DNA to RNA to protein is the cornerstone of modern molecular biology. Although this flow appears to be straightforward, there is a very complicated relationship between mRNAs and its encoded proteins. Besides of the regulatory networks in co-/post-transcription processes, protein quantity in cells is influenced by many aspects, including codon composition, ribosomal entry sites, translation rates, protein half-life, mRNA and protein degeneration rate, protein modulation, folding and transport rates^1^. With next-generation sequencing breakthroughs in recent years, increasing amounts of large-scale transcriptome and proteome data are now available, and researchers must disentangle the general rules governing protein production. Although the concept is still controversial^2,3^, several studies have characterized a significant positive correlation between mRNA and protein levels (rho > 0.5) in yeast, Drosophila, *Caenorhabditis elegans*, zebrafish, human and plants cells in the “steady” states^4–7^. Thus, mRNA levels are suggested to be the primary factor determining protein levels. Based on these calculations, mRNA levels possibly accounts for 40% to 80% of protein abundance variance; other factors, such as translation rate, play a relatively minor role in protein production^8,9^. However, recent observations from fission yeast, human tumors and evolutionary studies between humans and chimpanzees, revealed that fold changes in mRNA levels are generally larger than fold changes in protein levels^10–12^. This phenomenon is termed as protein homeostasis that protein production is maintained at a stable level in the cell despite fluctuations in mRNA^1^. Two concepts reveal the complicated nature of the protein production process: the first stresses the importance of mRNA quantity in determining protein production, whereas the second indicates that other regulatory mechanisms are also important in determining a stable protein concentration. Thus, further studies, especially in different species or under different cellular conditions such as environmental pressures, are needed to elucidate protein homeostasis.

Mycotoxins are a group of low-molecular-weight secondary metabolites produced by filamentous fungi^13^. Approximately 400 mycotoxins have been identified and a dozen have been recognized as important threats to humans and animals, including aflatoxin, citrinin, ergot alkaloids, fumonisin B_1_, ochoratoxin A (OTA), patulin, trichothecenes, zearalenone (ZEN) and so on^14^. Mycotoxin contamination in crops is widespread around the world, especially in developing countries due to poor preharvest practice and postharvest storage and transportation^15^. It is estimated that 25% of world’s crop may be contaminated by mycotoxins. The US Council for Agricultural Science and Technology estimated that the cost of crop losses from aflatoxins, fumonisins and deoxynivalnol is $932 million USD per year and mitigation cost is $466 million per year^16^. Some mycotoxins have stable molecular properties which are difficult to be removed by common practices such as heating or filtering, and as the consequence, consumptions of mycotoxin-containing foods and feeds lead to various pathologic reactions and can even increase mortality rates^13,17,18^.

Aflatoxin B_1_ (AFB_1_), OTA and ZEN are some of the most widely spread mycotoxins in the world. Previous studies reported these mycotoxins have similar toxic actions in cells including prohibiting RNA and protein synthesize, DNA damage and ROS^13,18,19^, but none of them quantified the expression profile at the omics level. In this study, we sequenced the entire transcriptome and proteome of chicken (*Gallus gallus*) primary hepatocytes under AFB_1_, OTA and ZEN treatments. As in steady-state cells, the protein homeostasis pattern was preserved under mycotoxin pressure. However, genes from different pathways showed differing levels of protein homeostasis, indicating protein homeostasis was primarily maintained by housekeeping pathways in the cell.

## Results

### Mycotoxins treatment of chicken primary hepatocytes

**Figure 1:**
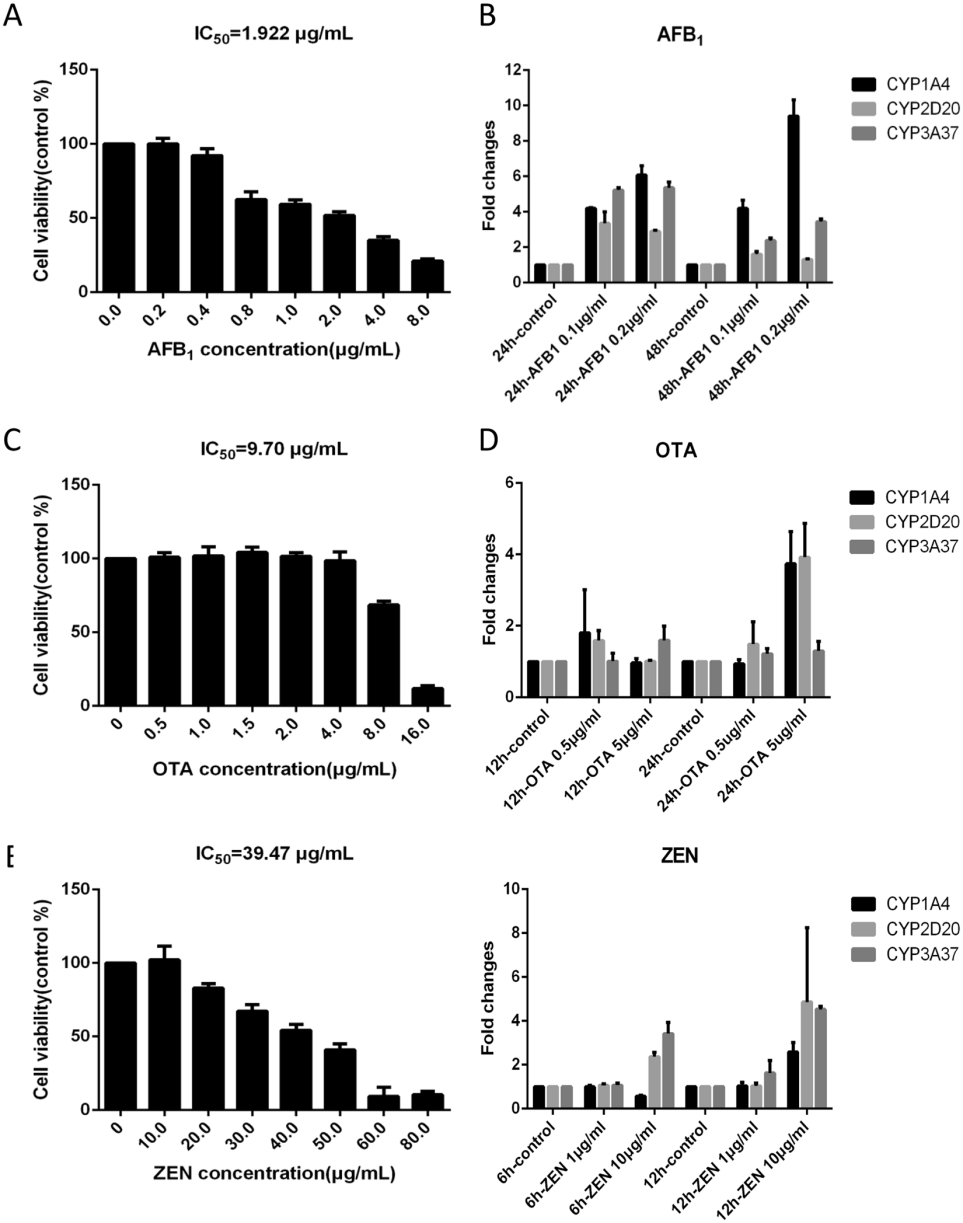
Chicken hepatocytes cell viability from MTT assay under (**A**) AFB_1_, (**C**) OTA and (**E**) ZEN administration. Each bar represents mean cell viability from five independent experiments with standard deviation. The gradient test for CYP1A4, CYP2D20 and CYP3A37 genes expression under different (**B**) AFB_1_, (**D**) OTA and (**F**) ZEN concentration and duration. Each bar represents mean fold changes from three independent experiments with standard deviation.

Chicken primary hepatocytes were administrated by three mycotoxins AFB_1_, OTA and ZEN. We used an MTT assay to assess cell viability under mycotoxin administration. The mycotoxin concentration was lower than 0.5 μg/mL, 5 μg/mL and 20 μg/mL (AFB_1_, OTA and ZEN) to maintain cell viability >90% (Fig. 1). Then, a gradient test was conducted to quantify CYP1A, CYP2D and CYP3A gene expression under different mycotoxin concentration and duration (Fig. 1). We used the lowest and shortest toxin treatment that induced the expression of all three CYP450s, which were 0.1 μg/mL 24 h for AFB_1_, 5 μg/mL 24 h for OTA and 10 μg/mL 12 h for ZEN, to administrate the chicken primary hepatocytes.

### High-throughput sequencing

Three biological replicates were collected for transcriptome and proteome sequencing from both untreated hepatocytes as the control, and mycotoxin-treated hepatocytes. To analyze the transcriptome, ~50 million reads (150-bp paired-end reads generated by Illumina, 6.34 to 7.52 Gb in total) were obtained for each replicate. High-quality scores (Q20 > 95%), low error rates (0.02%) and stable GC content indicated a high-quality transcript dataset (Table S1). To verify the data consistency among biological replicates, we applied Pearson’s correlation analysis between all samples (Fig. S1). The correlations were all >0.98, higher than the best practice guideline by ENCODE Consortium (0.92–0.98).

For the proteome analysis, we used iTRAQ (isobaric tag for relative and absolute quantification) technology to quantify relative protein levels between three mycotoxin-treated and control samples. Spectra from tandem mass spectrometry were searched using MASCOT engine 2.2 (Matrix Science, London UK) against the UniProt chicken database for peptide identification (https://www.ebi.ac.uk/GOA/chicken_release, Uniprot_Chicken_24083_20150713.fasta). Peptides were quantified by Proteome Discoverer 2.0 (Thermo Fisher Scientific, San Jose, CA) and the false discovery rate (FDR) was calculated based on a Decoy database search with a cutoff of 0.01. The majority of peptide lengths ranged from 5 to 25 amino acids (Fig. S2), similar to the range reported previously^20^. Protein ratios were calculated based on the combined median ratio of unique peptides, and experimental bias was controlled by normalizing the median protein ratio to 1. The final protein ratio was the mean of the three biological replicates (Table S2).

### Differential expressed transcripts and proteins

**Figure 2:**
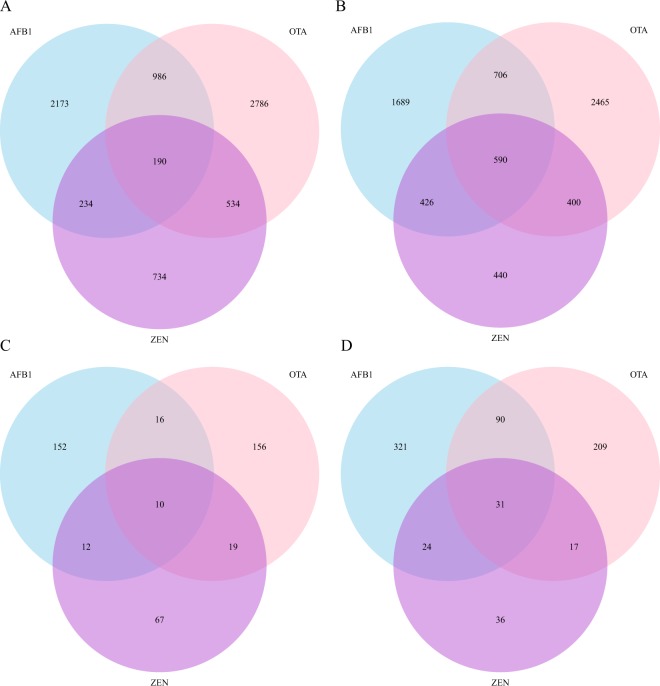
Ven diagram plot for the (**A**) up-regulated and (**B**) down-regulated transcripts and (**C**) up-regulated and (**D**) down-regulated proteins among three mycotoxins.

**Figure 3:**
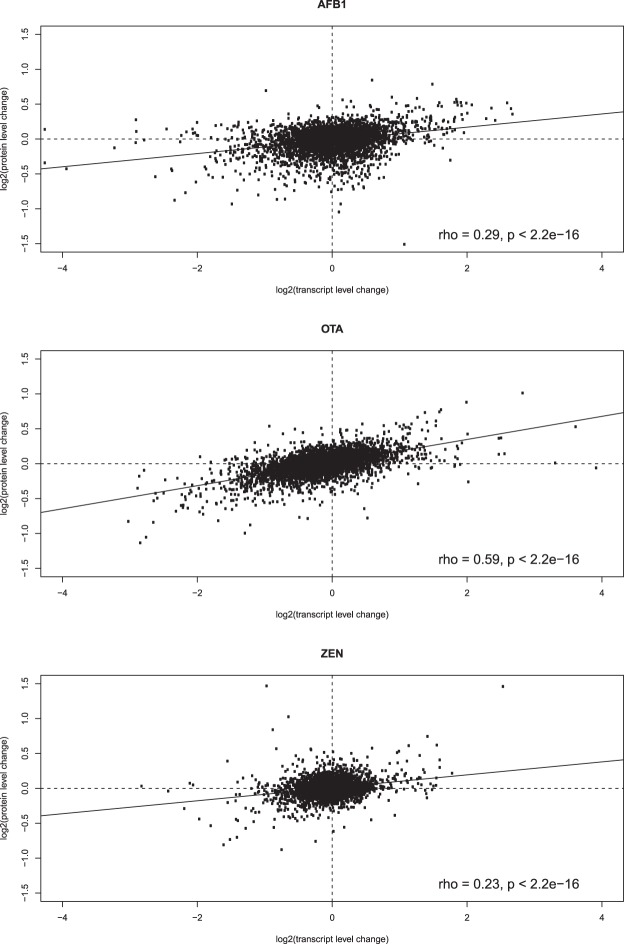
Pearson’s correlation between transcript and protein level changes for AFB_1_, OTA and ZEN. Transcript and protein changes are calculated by comparing mycotoxin treated samples and control samples.

The set of differentially expressed transcripts (DETs) were first characterized. We got quantitative expression level for 19,948 transcripts in total, and the DETs were identified based on negative binomial distribution with Benjamini and Hochberg procedure adjusted p value < 0.05^21^. Compared to the control treatment, mycotoxin treatment led to intensive transcriptomic changes (Figs 2 and S3). For AFB_1_, we found 6,994 DETs (35.1% of total transcripts), including 3,583 up-regulated and 3,411 down-regulated transcripts. For OTA, there were 8,657 DETs (43.4% of total transcripts), including 4,496 up-regulated and 4,161 down-regulated genes. For ZEN, there were 3,548 DETs (17.8% of total transcripts), including 1,692 up-regulated and 1,856 down-regulated genes (Table S3; Fig. 3). The number of shared up-regulated and down-regulated transcripts among all three mycotoxins was much lower than the mycotoxin-specific DETs, especially for up-regulated transcripts (Fig. 2A,B). We further grouped the up-regulated and down-regulated transcripts based on the KEGG pathway definition (Tables 1 and 2). For AFB_1_ treated samples, the up-regulated transcripts were mainly enriched in the “Environmental information processing” pathway, including “Cytokine-cytokine receptor interaction”, “ECM-receptor interaction”, “Jak-STAT signaling” and “Cell adhesion molecules (CAMs)” pathways. For ZEN treated samples, the up-regulated transcripts were mainly enriched in “Genetic information processing” pathway, including “DNA replication”, “Spliceosome”, “RNA transport”, “Mismatch repair”, “Homologous recombination”, “Base excision repair” and “RNA polymerase” pathways. The down-regulated transcripts were enriched in “Metabolism” and “Cellular processes” pathways for AFB_1_, “Ribosome” and “Focal adhesion” pathways for OTA and “Valine, leucine and isoleucine degradation” and “PPAR signaling” pathways for ZEN.

**Table 1:** KEGG pathways with significant enriched up-regulated transcripts and proteins.

Toxin	KEGG pathway (transcriptome)	P value	KEGG pathway (proteome)	P value
AFB_1_	Cytokine-cytokine receptor interaction	0.02	Fructose and mannose metabolism	0.00013
	Intestinal immune network for IgA production	0.001739	Retinol metabolism	0.000283
	Jak-STAT signaling pathway	0.003656	Chemical carcinogenesis	0.002798
	Glycosaminoglycan biosynthesis - chondroitin sulfate/dermatan sulfate	0.003893	Biosynthesis of amino acids	0.004614
	ECM-receptor interaction	0.007142		
	Phagosome	0.007149		
	Cell adhesion molecules (CAMs)	0.008871		
OTA			Complement and coagulation cascades	0.000614
ZEN	Cell cycle	2.72E-12	Biosynthesis of amino acids	0.001971
	DNA replication	6.67E-11		
	Fanconi anemia pathway	5.08E-09		
	Ribosome biogenesis in eukaryotes	4.35E-08		
	Pyrimidine metabolism	9.56E-08		
	Spliceosome	6.70E-07		
	RNA transport	1.06E-06		
	Mismatch repair	8.52E-06		
	Homologous recombination	9.64E-06		
	Nucleotide excision repair	0.00104		
	Base excision repair	0.001211		
	RNA polymerase	0.001602		
	mRNA surveillance pathway	0.002268		
	Purine metabolism	0.005707		
	p53 signaling pathway	0.008001		

P value is calculated by Fisher’s exact test with FDR correction.

**Table 2:** KEGG pathways with significant enriched down-regulated transcripts and proteins.

Toxin	KEGG pathway (transcriptome)	P value	KEGG pathway (proteome)	P value
AFB_1_	Metabolic pathways	0.000886	ECM-receptor interaction	8.14E-07
	Peroxisome	0.004771	Cell adhesion molecules (CAMs)	4.79E-05
	Fatty acid metabolism	0.00858	Focal adhesion	8.65E-05
	Cell cycle	0.009167	Axon guidance	1.01E-04
			Ribosome	1.36E-04
			Ribosome biogenesis in eukaryotes	3.28E-04
			Complement and coagulation cascades	7.82E-04
			Arrhythmogenic right ventricular cardiomyopathy (ARVC)	1.45E-03
			Cytokine-cytokine receptor interaction	2.25E-03
			Small cell lung cancer	2.89E-03
			Hippo signaling pathway	4.02E-03
			Fatty acid biosynthesis	4.09E-03
			Adherens junction	8.27E-03
			Pathways in cancer	8.63E-03
			Proteoglycans in cancer	9.43E-03
OTA	Ribosome	1.41E-06	ECM-receptor interaction	1.04E-06
	Focal adhesion	0.00217	Focal adhesion	4.83E-05
			Amoebiasis	8.06E-04
			Small cell lung cancer	4.59E-03
			PI3K-Akt signaling pathway	7.94E-03
ZEN	Valine, leucine and isoleucine degradation	0.003184	Insulin signaling pathway	0.008517
	PPAR signaling pathway	0.003962		

P value is calculated by Fisher’s exact test and cutoff set to 0.01.

We identified much less number of differentially expressed proteins (DEPs): only 656 (10.3%), 548 (8.6%) and 216 (3.4%) DEPs were between AFB_1_-, OTA- and ZEN- treated and control samples (Table S3; Figs 2 and S4). Similar to the transcript dataset, the number of shared DEPs was much lower than the mycotoxin-specific DEPs (Fig. 2). For AFB_1_ treated samples, the up-regulated proteins were enriched in “Metabolism” pathway, including “Fructose and mannose metabolism”, “Retinol metabolism” and “Biosynthesis of amino acids” pathways, and cancer related “Chemical carcinogenesis” pathway. For ZEN, the up-regulated proteins were enriched in “Biosysthesis of amino acids” pathway, and for OTA in “Complement and coagulation cascades” pathway (Table 1). For AFB_1_ treated samples, the down-regulated proteins were enriched in “Environmental information processing” pathway, including “ECM-receptor interaction”, “Cell adhesion molecules” and “Cytokine-cytokine receptor interaction”, and pathways from cellular community and cancer. For OTA treated samples, the down-regulated proteins were enriched in “ECM-receptor interaction” pathway, and for ZEN in “Insulin signaling pathway”.

Although we found some similarities between different mycotoxin administrations, such as “ECM-receptor interaction” pathways were enriched with down-regulated proteins for both AFB_1_ and OTA, the overall distribution of DETs and DEPs was quite different for the three mycotoxins. Furthermore, the set of DETs were not consistent with the set of DEPs (Tables 1 and 2). For AFB_1_, seven pathways were enriched with up-regulated transcripts but none of them was enriched with up-regulated proteins. Similarly, four pathways were enriched with up-regulated proteins but none of them was enriched with up-regulated transcripts. This pattern suggests that each pathway may assume a specific mRNA and protein production dynamics under mycotoxin pressure.

### Protein homeostasis under mycotoxin stress

We extracted 4,110 genes with quantitative transcript (FPKM > 1) and protein expression values for all three mycotoxins, and visualized the expression fold changes (Fig. 3). We found a weak significant positive correlation between the transcript and protein changes for all mycotoxins (p < 2.2e-16), and the correlations for AFB_1_ (0.29) and ZEN (0.23) were lower compared to previous reports for cells in the “steady” states (rho > 0.5). The transcript level changes were much higher than protein level changes for most genes, thus the protein homeostasis pattern was preserved under mycotoxin stress (Fig. 2).

**Figure 4:**
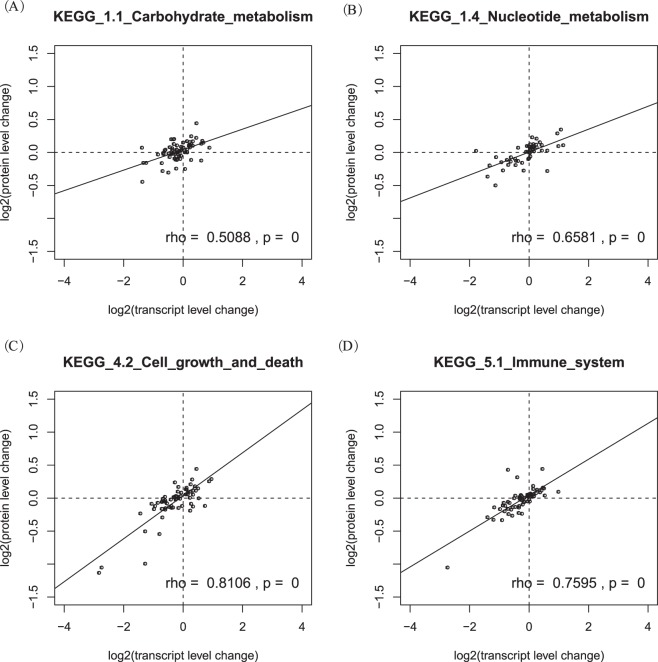
Pearson’s correlation between transcript and protein level changes for four representative KEGG pathways in OTA treated samples. (**A**) Carbohydrate metabolism; (**B**) Nucleotide metabolism; (**C**) Cell growth and death; and (**D**) Immune system. Transcript and protein changes are calculated by comparing mycotoxin treated samples and control samples.

We further plotted transcript and protein changes for each KEGG pathway for AFB_1_, OTA and ZEN (Figs S5–S7). We found certain pathways, such as “Carbohydrate metabolism” and “Nucleotide metabolism” under OTA, showed lower protein /mRNA changes than the “Signal transduction” and “Cell growth and death” pathways (Fig. 4). More specifically, we used slope (the fitted line for linear regression) to represent the overall protein/mRNA fold changes. A higher slope indicated higher protein-to-mRNA changes and relaxed constraint on protein homeostasis. We listed the complete results in Table S4 and summarized the results in Table 3. To be conservative, we only showed pathways with correlations >0.3 and p values < 0.01. As most housekeeping genes are associated with the “Metabolism”, “Transcription” and “Translation” pathways (KEGG 1.1 to 2.2) and most genes in other pathways (KEGG 2.3 to 5.3) are non-housekeeping genes^22^, we classified the two sets of pathways as housekeeping pathways and non-housekeeping pathways. In OTA-treated samples, housekeeping pathways showed lower protein/mRNA changes than non-housekeeping pathways (p = 0.001, two-tailed t-test). In AFB_1_-treated samples, the overall pattern was the same (p = 0.044). In ZEN-treated samples, we did not observe higher changes in non-housekeeping than housekeeping pathways. The slopes for non-housekeeping pathways were still higher than the slopes of most housekeeping pathways, but the pattern was distorted by the relatively high slope for “Energy metabolism” (0.293). Combining the results obtained with all three mycotoxins, we identified several pathways with high protein changes, including “Cell growth and death” (>0.2 for all three mycotoxin), “Folding, sorting and degradation”, “Replication and repair”, “Membrane transport”, and “Immune system” (>0.2 for two mycotoxin).

**Table 3:** Correlation and linear regression slopes between protein and transcript level changes for KEGG pathways for AFB_1_-, OTA- and ZEN-treated samples.

KEGG pathways	AFB_1_		OTA		ZEN		Datasize No.gene
	rho	slope	rho	slope	rho	slope	
Carbohydrate metabolism	0.332	0.104	0.51	0.155			69
Energy metabolism			0.44	0.147	0.672	**0.293**	37
Lipid metabolism	0.486	0.171	0.45	0.152	0.359	0.114	77
Nucleotide metabolism	0.391	0.119	0.66	0.174			51
Amino acid metabolism	0.407	0.13	0.63	0.181	0.478	0.144	60
Glycan biosynthesis and metabolism	0.395	0.122	0.41	0.12	0.495	0.178	57
Metabolism of cofactors and vitamins			0.52	0.152			35
Transcription			0.51	0.149			48
Translation			0.48	0.131	0.339	0.149	100
Folding, sorting and degradation			0.66	**0.209**	0.526	**0.222**	111
Replication and repair	0.474	0.176	0.73	**0.29**	0.464	**0.222**	25
Membrane transport	0.926	**0.221**	0.9	**0.468**			11
Signal transduction	0.325	0.141	0.7	**0.259**	0.412	0.185	186
Signaling molecules and interaction			0.57	**0.203**	0.462	0.177	137
Transport and catabolism	0.388	0.134	0.65	**0.241**			126
Cell growth and death	0.497	**0.221**	0.81	**0.326**	0.602	**0.238**	65
Cellular community - eukaryotes	0.42	0.12	0.49	0.169			108
Cell motility			0.66	**0.307**			42
Immune system	0.412	0.144	0.76	**0.272**	0.515	**0.271**	60
Endocrine system			0.7	**0.211**	0.276	0.109	103
Circulatory system	0.572	**0.226**	0.57	0.148			44

We only present pathways with data sizes (number of genes) larger than 10, correlation values above 0.3 and p value less than 0.01. Slopes greater than 0.2 are defined as high protein/mRNA changes and formatted with bold font.

Overall, we found variable protein homeostasis pattern under mycotoxin administration. It is tempting to hypothesize a fitness optimization process in cell that the strong constraint on housekeeping pathways is a baseline requirement for the maintenance of cellular function, and the relaxed constraint on non-housekeeping pathways is primarily related to the functional response to specific mycotoxin pressures.

### Other factors associated with protein homeostasis

**Figure 5:**
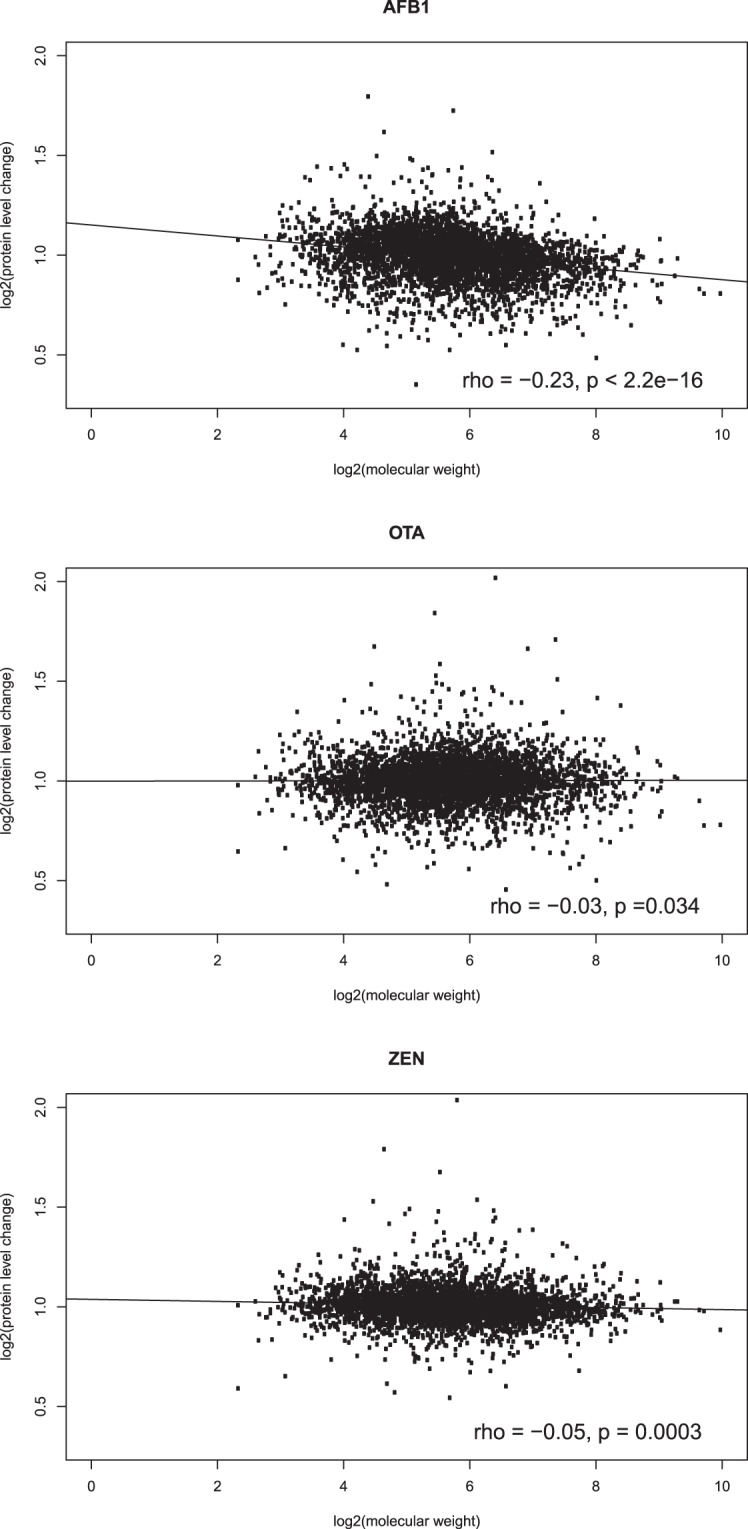
Pearson’s correlation between protein molecular weight and protein level changes under (**A**) AFB_1_, (**B**) OTA and (**C**) ZEN treatment.

Molecular weight may be involved in protein homeostasis. It has been hypothesized that protein production cost is primarily determined by protein molecular weight, and thus, the overproduction of a low-cost protein should have a small fitness effect, whereas the production of a high-cost protein is more tightly controlled^23^. Thus, the homeostasis of proteins with high molecular weights should be stronger than the homeostasis of low-molecular-weight proteins. To test this assumption, we calculated the correlation between molecular weight and protein level changes in the three mycotoxin-treated samples, and we found very weak but significant negative correlations for all three samples (rho −0.029, −0.054 and −0.23, p < 0.05; Table 4, Fig. 5). This result suggests that molecular weight may only play a minor role or be relevant for a subset of proteins. We further conducted the analyses on housekeeping and non-housekeeping pathways separately, and found a negative correlation for each analysis (Table 4). The correlation was higher for non-housekeeping pathways than for housekeeping pathways under AFB_1_ and OTA treatment. For ZEN treatment, the correlations and p values were similar between the two sets. This result suggests that molecular weight affects both gene sets, but the effect is stronger for non-housekeeping pathways, consistent with the hypothesis of a relaxed protein homeostasis constraint on these gene sets.

**Table 4:** Correlation between molecular weight and protein level changes.

Toxin	All data		Housekeeping gene		Non-housekeeping gene	
	rho	p value	rho	p value	rho	p value
AFB_1_	−0.23	0	−0.24	6.83E-08	−0.27	1.47E-12
OTA	−0.029	0.034	−0.046	0.16	−0.11	0.0034
ZEN	−0.054	0.00032	−0.069	0.064	−0.064	0.051

Protein location may also be involved with protein homeostasis. We used MetazSecKB^24^ to predict chicken protein locations and evaluate the impact of cellular location on protein changes (Tables S5 and S6; Figs S8–S10). In general, we found that proteins associated with organelle membranes showed higher protein changes than secreted, cell plasma membrane-associated and in-lumen proteins (p = 0.02, two-tailed t-test). In particular, “Golgi apparatus membrane” and “Nuclear membrane” genes had high slopes (>0.2), and genes in the “Cytoplasm” location had low slope values. The data suggests that subcellular location is also associated with protein homeostasis under mycotoxin stress. However, gene function and location are closely confounding factors. For example, membrane proteins primarily function as signal transduction receptors, substrate transporters and enzymes^25^, and the majority of metabolism and information processing genes are located in the cytoplasm. Thus, it is necessary to take into account of the two features together and interpret the results with caution.

### Long non-coding transcript isoform is not the mechanism for protein homeostasis

Previous studies reported that the length of 5′ UTRs influenced mRNA translation efficiency; the isoform of long undecoded transcript had poor translational rate compared to the canonical transcripts^26^. We analyzed the expression of long UTRs and normal UTRs in the mycotoxin-treated and control samples. The long UTRs were defined as UTRmycotoxin – UTRcontrol > 500 bp, and normal UTRs were defined as −5 < UTRmycotoxin – UTRcontrol < 5. Only UTRcontrol < 600 bp were included in the analysis^27^. We did not find association between the UTR lengths and protein expression levels in our dataset (Fig. S11). Thus, UTR length variation is not the mechanism for protein homeostasis in chicken hepatocytes.

## Discussion

In this study, we used widespread mycotoxins AFB_1_, OTA and ZEN to administrate chicken embryo hepatocytes and characterize the transcriptomic and proteomic changes. The high-throughput data indicated that the three mycotoxins induced variable sets of differential expressed transcripts and proteins, but the overall pattern of protein homeostasis was still preserved. This study systematically evaluated the association between protein homeostasis and molecular factors, including gene function, protein location and molecular weight, and hypothesis a fitness optimization process in cell.

AFB_1_ is one the most studied and characterized mycotoxin to date due to the outbreak of turkey X disease in the early 1960s^28^. It is well established that active metabolites of AFB_1_ can bind to the N7 position of guanines, and the AFB_1_-DNA adducts result in GC to TA transversions^13^. Other biochemical actions include decreasing RNA content and RNA polymerase, suppressing protein synthesis, interrupting carbohydrate metabolism by decreasing glycogen level and inhibiting electron transport in mitochondria^29^. Similarly, OTA also has multiple biochemical actions in cell, including oxidative stress, protein synthesis inhibition and activation or deactivation of specific cell signaling pathways^30^. ZEN and its derivatives can competitively bind to the estrogen receptor, leading to various estrogenic syndromes^13^. Study on cultured Vero cells reveals that ZEN and its derivatives also inhibit protein and DNA synthesis, cell viability, oxidative damages and over-expression of stress proteins^31^. Thus, all three mycotoxins have multiple biochemical and molecular actions in cell, but it is unclear whether they affect the same set of genes/pathways or not. In this study, we found that each mycotoxin had quite specific impact on functional pathways, led to different molecular action and cellular responses in chicken hepatocytes. The phenomenon are unlikely due to different stress levels posed by these mycotoxins. We controlled the mycotoxin stress on the minimum level to maintain >90% cell viability throughout the study. The lowest level of mycotoxin concentration and shortest duration time that induced the expression of CYP450s have been chosen to administrate the chicken cells. Thus, stress levels should not be a major factor leading different cellular responses.

Previous studies reported that the iTRAQ technique underestimated the fold change signals by compressing the isotopic intensity ratios and the underestimation became more obvious for proteins with high changes^32,33^. Another study estimated the iTRAQ measurement had ~40–50% compression to the true value^34^. In our study, the changes of protein expression was several fold lower than the transcript expression, thus the technique compression can contribute to the fold differences, but should not be a major explanation for the protein homeostasis pattern we observed. Another potential problem is that protein production is slower than mRNA production, and thus, the protein homeostasis we observed may be attributable to a production delay rather than a true stasis effect. However, previous studies showed that protein production was generally only delayed for 5 or 6 h after the mRNA oscillations^9,35^. In this study, we applied AFB_1_ and OTA treatment for 24 h and ZEN for 12 h, which are much longer times than the reported delay in protein production. Thus, the effects of “production delay” should be minimal in our study.

Protein production is much more expensive than mRNA production. Warner *et al*. estimated that cells divert 50% of their energy to protein production, whereas only 5% of energy is spent on mRNA production^36^. It costs 2 GTP and 1 ATP for each peptide bond formed, whereas only 1 NTP is required for nucleotide elongation. Researchers estimated that the total number of proteins is ~1,850- to ~10,000- fold higher than mRNA copy numbers in fission yeast and mammals^10,37,38^. Thus, protein production is very costly in term of cellular energy and resources but is crucial for maintaining cellular function. According to evolutionary theory, protein production should be maximized or constrained according to the fitness of a cell or organism, which is termed as an optimization process^39,40^. This theory has been tested for a few phenotypes and genes, such as the production of Lac protein in *Escherichia coli*, but has never been tested at the whole-transcriptome/proteome level. The pathway-specific protein homeostasis observed in this study fit this evolutionary optimization process and demonstrated selective advantages for cell survival under mycotoxin pressure. Genes involved in specific functions such as “Cell growth and death” or “Immune system” had relaxed constraints on protein production, demonstrating clear advantages for active responses to the changing environment. However, the housekeeping pathways experienced a stronger constraint to maintain much more stable protein concentrations for baseline cellular functions. By maintaining stable concentrations, disruptive signals from toxic molecules do not interfere with core gene production, which in turn reduces toxic cellular effects in response to relatively minor damage.

Homeostasis theories can be soundly described and explained by this cost-benefit optimization theory, but the molecular mechanism underlying buffering remains elusive. Researchers have tried to explain the pattern from the perspective of translational rates but have found conflicting results. Some evidence suggests that translation rates contribute significantly to protein buffering^41,42^, whereas others studies have indicated that translation rates are not important^43,44^. Dephoure *et al*. reported that translation rate variation was not important for the large-scale regulation of protein production; however, the protein degradation rate may be a major factor shaping protein homeostasis patterns^45^. A recent proteomic study in mouse cardiac cells also supported this hypothesis by revealing that functionally associated protein were co-regulated in degradation process^46^. Others studies alternatively proposed that the microRNAs may contribute significantly to mRNA and protein correlations^47,48^. A new hypothesis suggested that variation of mRNA length altered the meiotic protein expressions levels^26^. We measured the expression profile for transcripts with long and normal UTRs and failed to find any association in the chicken model (Fig. S7). But as only a small portion of long non-coding RNAs were identified in our study due to the sequencing depth limitation, we cannot totally rule out this possibility. Overall, it is unclear what is the main mechanism to buffer the protein variation and whether different organisms have same mechanism or not, thus more studies are needed in the future to address these issues.

## Methods

### Experimental design and statistical rationale

In this study, we performed transcriptome and proteome sequencing for three mycotoxin-treated samples (AFB_1_, OTA and ZEN) and one control sample. For each sample, we collected and sequenced three biological replicates. To detect differentially expressed genes, Benjamini and Hochberg’s approach was used to control the FDR. Pearson’s correlation was used for correlation analysis because the dataset has a normal distribution. We included all data points in the statistical analyses.

### Chicken primary hepatocytes isolation and mycotoxin preparation

Eighteen-day embryonated specific-pathogen-free (SPF) Arbor Acres Broiler chicken eggs were purchased from the Institute of Animal Science, Guangdong Academy of Agricultural Sciences (Guangzhou, China). All related experiments and methods were performed in accordance with the recommended guidelines and regulations by the Administration of Affairs Concerning Experimental Animals of Guangdong Province, China. This research was approved (approval number 2015-D010) by Laboratory Animal Ethics Committee of South China Agricultural University. Hepatocytes were prepared using a collagenase digestion method^49^. Chicken embryo primary hepatocytes were re-suspended in Williams E medium (Sigma-Aldrich, Shanghai) containing 10% FBS, 100 U/mL penicillin/streptomycin, 10 nM insulin, and 10 nM dexamethasone (Sigma-Aldrich). We added the medium to each culture plate and cultured cells at 37 °C in a humidified incubator with 5.0% CO2. After 5 h, the medium was removed from adherent cells, and the cells were maintained in Medium 199 (Life Technologies, Shanghai) supplemented with 10% FBS, 100 U/mL penicillin/streptomycin, 10 μg/mL insulin, 1 μg/mL dexamethasone, and 2 mM L-glutamate (Invitrogen). AFB_1_, ZEN and OTA were purchased from Pribolab (Qindao, China). All mycotoxins were dissolved in Dimethyl sulfoxide (DMSO). Williams E medium, Medium 199 and FBS were purchased from Invitrogen (Carlsbad, CA, USA).

### MTT cell viability assay and quantitative RT-PCR

Cell viability was assessed according to a previously described method^50^. Chicken embryo primary hepatocytes were seeded at a density of 10000 cells/well in 96-well plates and cultured overnight. The hepatocytes were treated with different concentrations of AFB_1_ (0–10 μg/mL), ZEN (0–120 μg/mL), or OTA(0–10 μg/mL). After 48 h, 0.5 mg/mL MTT (Sigma-Aldrich, Shanghai) was added to each well. We extracted the total RNA from mycotoxin-treated or untreated chicken primary hepatocytes using Trizol reagent (Invitrogen, Carlsbad, CA, USA). Total RNA (2 μg) was reverse transcribed using M-MLV reverse transcriptase (Promega, Madison, WI, USA) with oligo d(T) and random primers (Takara Biotechnology, Dalian, China). The obtained cDNA products were used as templates for CYP1A4, CYP2D20 and CYP3A37 transcript quantification by RT-PCR. GAPDH was used as an internal reference to normalize gene expression. The expression levels were calculated using the 2−ΔΔCt method^51^.

### Library preparation and transcriptome sequencing

Total RNA purity, concentration and integrity were assessed using NanoPhotometer® spectrophotometry (IMPLEN, CA, USA), Qubit® RNA Assay Kit in a Qubit® 2.0 Fluorometer (Life Technologies, CA, USA), and RNA Nano 6000 Assay Kit on a Bioanalyzer 2100 system (Agilent Technologies, CA, USA). A total of 3 μg of RNA was extracted per sample. Sequencing libraries were constructed using NEBNext® Ultra™ RNA Library Prep Kit for Illumina® (NEB, USA), then sequenced on an Illumina Hiseq X Ten platform, with paired-end reads length of 150 bp.

### Transcriptome data analysis

Raw data were processed through Perl scripts by removing reads that contained adapter, ploy-N and low quality reads. The Q20, Q30 and GC contents for each sample were calculated. All downstream analyses were based on the processed clean data. The reference chicken genome and annotation files were downloaded from NCBI with the GenBank Assembly ID GCA_000002315.3. The index of the reference genome was built using Bowtie v2.2.3^52^, and paired-end clean reads were aligned to the reference genome using TopHat v2.0.12^53^. The read numbers mapping to each gene were calculated by HTSeq v0.6.1^54^, and the FPKM of each gene was calculated based on the length of the gene and the reads count mapped to that gene. Differential expression analysis was performed using the DESeq R package (1.18.0)^21^, and the statistical enrichment of the differential expression genes in KEGG (http://www.genome.jp/kegg/) pathways was calculated by KOBAS^55,56^.

### Sample preparation, iTRAQ labeling and mass spectrometry

SDT buffer was used for the sample preparation as in the universal proteomic sample preparation protocol^20^. For SDS-PAGE separation, 20 µg of proteins was mixed with 5X loading buffer and boiled for 5 min. The proteins were separated on a 12.5% SDS-PAGE gel (constant current 14 mA, 90 min) and visualized using Coomassie Blue R-250 staining. The peptide mixture of each sample (100 μg) was labeled with iTRAQ reagent according to the manufacturer’s instructions (Applied Biosystems, Shanghai). The labeled peptides were fractionated by SCX chromatography (GE Healthcare). LC-MS/MS analysis was performed with a Q Exactive mass spectrometer (Thermo Scientific, Shanghai) and Easy nLC (Proxeon Biosystems, Danmark). MS data was collected based on the most abundant precursor ions from the survey scan (300–1800 m/z).

### Proteome data analysis

MS/MS spectra were analyzed using MASCOT engine (Matrix Science, London, UK; version 2.2) with following parameter settings. The search was MS/MS ion search. The protease used to generate peptides was trypsin. Two missed cleavages were permitted. The mass values were monoisotopic. The list of all fixed modifications considered included carbamidomethyl (C), and iTRAQ4PLEX (N-terminal), iTRAQ4plex (K). The list of all variable modifications included oxidation (M) and iTRAQ4plex (Y). The peptide mass tolerance for precursor ions was ±20 ppm. The mass tolerance for fragment ions was 0.1 Da. We used the Decoy database embedded in Proteome Discoverer 2.0 (Thermo Fisher Scientific) to calculate the FDR and set the cutoff to <0.01 for a peptide. The retrieved sequences were locally searched against the UniProt GOA database (chicken) (https://www.ebi.ac.uk/GOA/chicken_release, Uniprot_Chicken_24083_20150713.fasta) using NCBI **BLAST+** client software (ncbi-blast-2.2.28 + -win32.exe)^57^ for annotation. Protein ratios were calculated as the median of only the unique peptides for a protein. To exclude experimental bias, we normalized all peptide ratios using the median protein ratio. Protein quantification was calculated using the normalized spectral index (SI_N_)^58^. Technical repeats were accessed by performing a multivariate Pearson correlation analysis with R. To categorize genes into specific KEGG pathways, we blasted the annotated proteins against the online KEGG database^55^. Mapping and ID conversion were facilitated using R package ‘org.Gg.eg.db’ in Bioconductor. Downstream analysis and graphic plotting for transcriptomic and proteomic data were primarily performed using the Rstudio platform^59^.

## Supplementary information

SupplimentFiguresTables

## Data Availability

The proteomic raw data were deposited in the ProteomeXchange Consortium via the PRIDE partner repository under the dataset identifier PXD008961. The sequenced RNAseq raw data, processed read counts and FPKM file were deposited in GEO database of NCBI (Accession Number GES112862).
