# Supplementary material for: Variable protein homeostasis in housekeeping and non-housekeeping pathways under mycotoxins stress
Source: Sci Rep. 2019 May 24;9:7819. doi: 10.1038/s41598-019-44305-0 (PMC6534621; doi:10.1038/s41598-019-44305-0)
Supplement: Supplementary file 1 — SupplimentFiguresTables [file 41598_2019_44305_MOESM1_ESM.pdf]

Variable protein homeostasis in housekeeping and non-housekeeping pathways under mycotoxins stress

Yu Sun[1,2*], Jikai Wen[1,2*], Ruohong Chen[1,2], Yiqun Deng[1,2**]

This supplementary file contains figures and tables.

Supplementary figures:

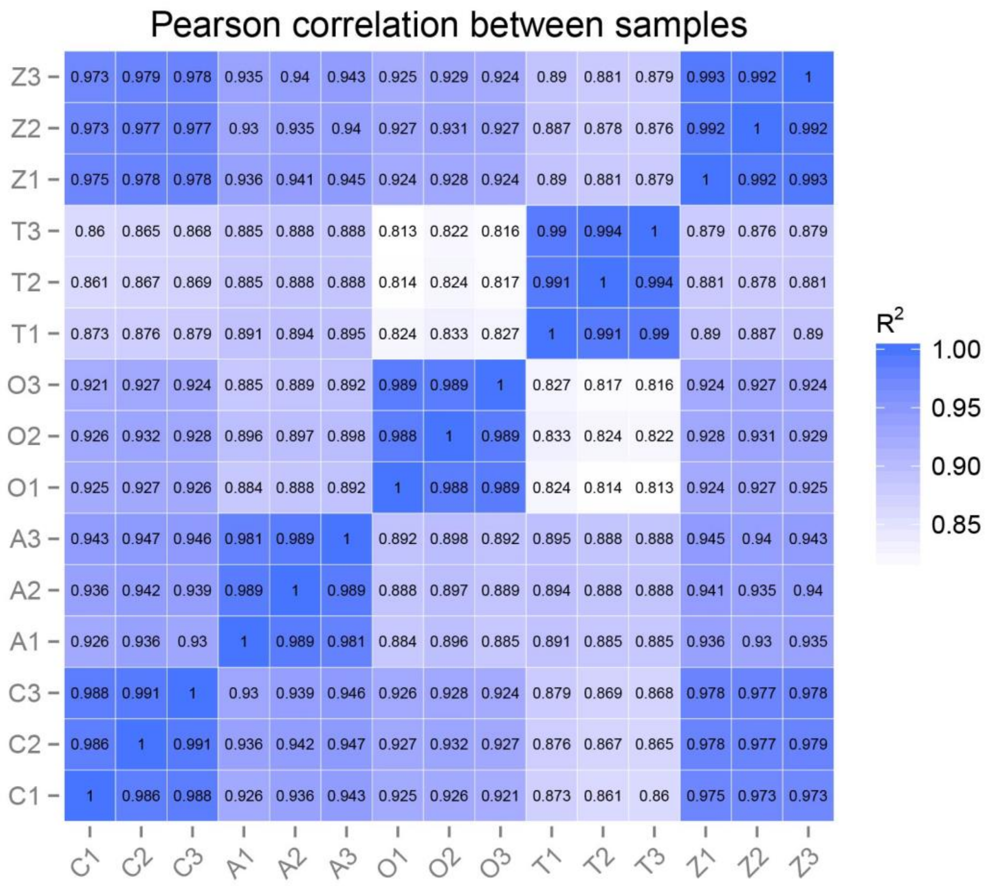

Figure S1. Pearson correlation between all biological replicates and samples for the RNAseq data.

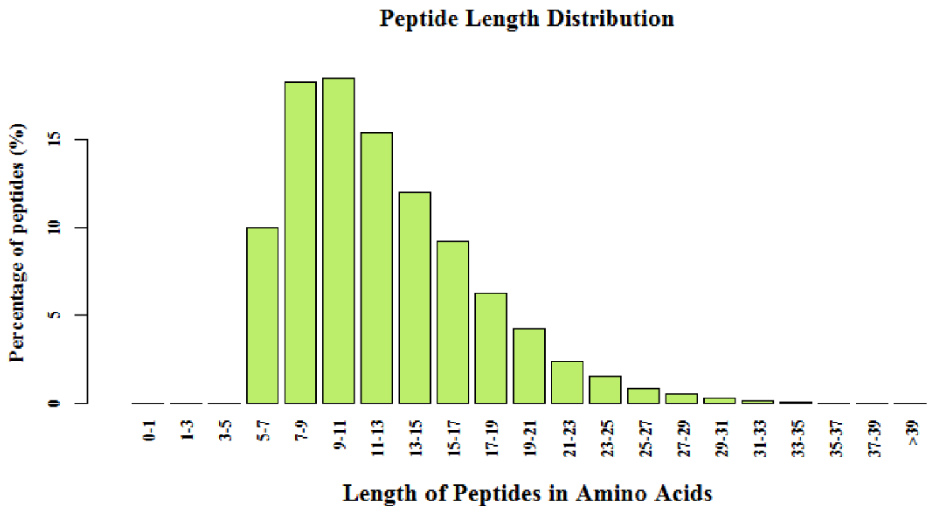

Figure S2. Peptide length distribution from iTRAQ analysis.

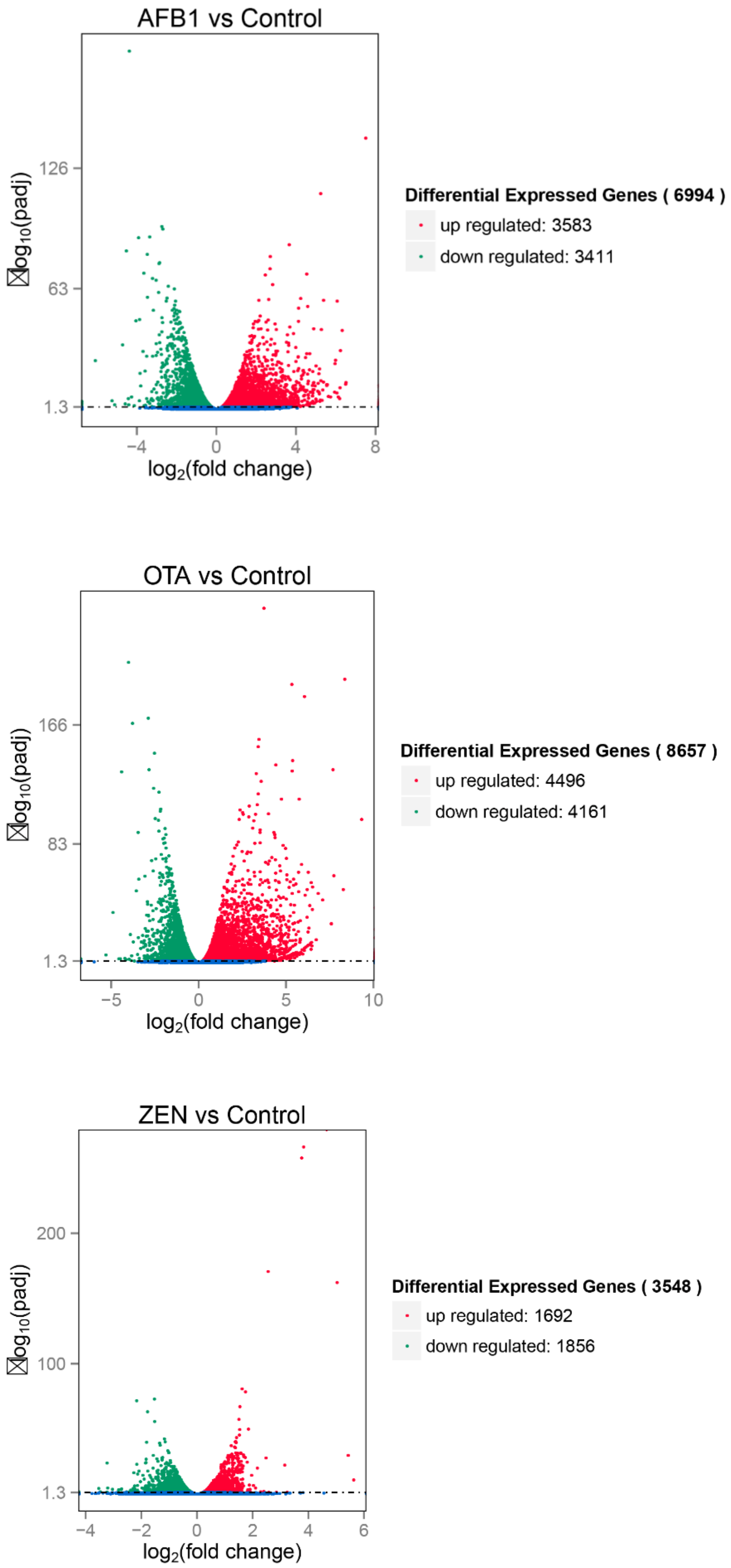

Figure S3. Volcano plot for the up-regulated and down-regulated differential expressed genes between (A) AFB_1 vs control (B) OTA vs control (C) ZEN vs control for the

transcriptional data.

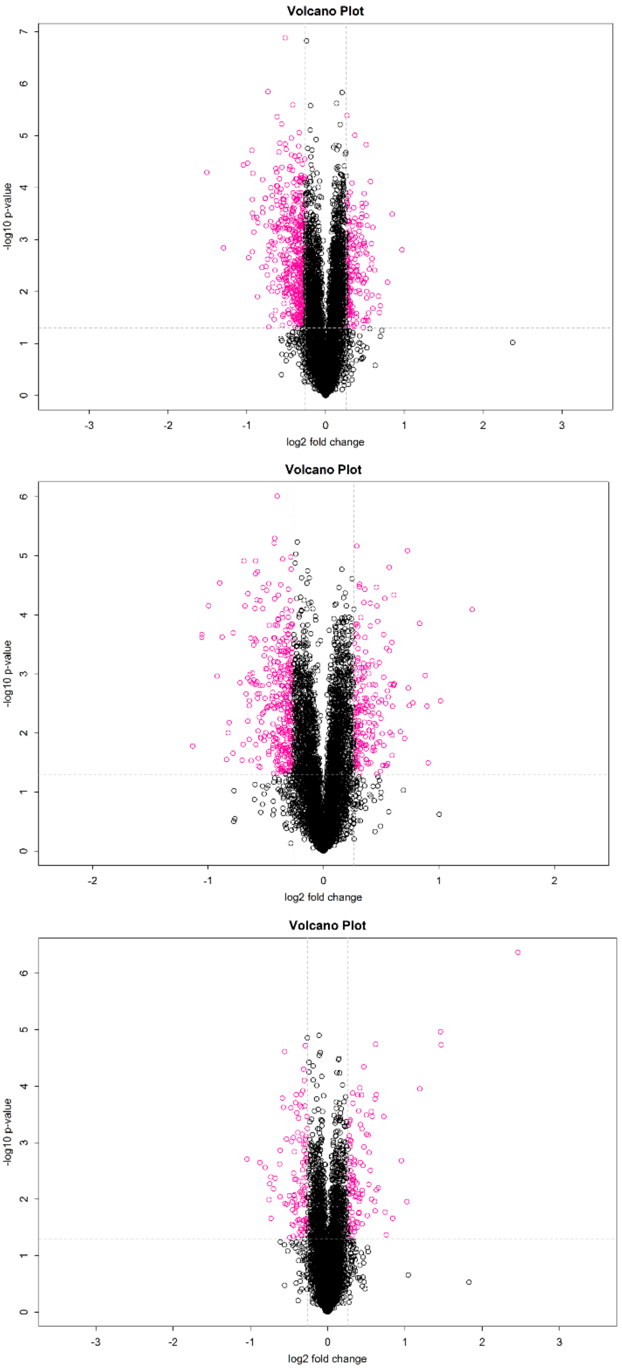

Figure S4. Volcano plot for the up-regulated and down-regulated differential expressed genes between (A) AFB_1 vs control (B) OTA vs control (C) ZEN vs control for the

proteomic data.

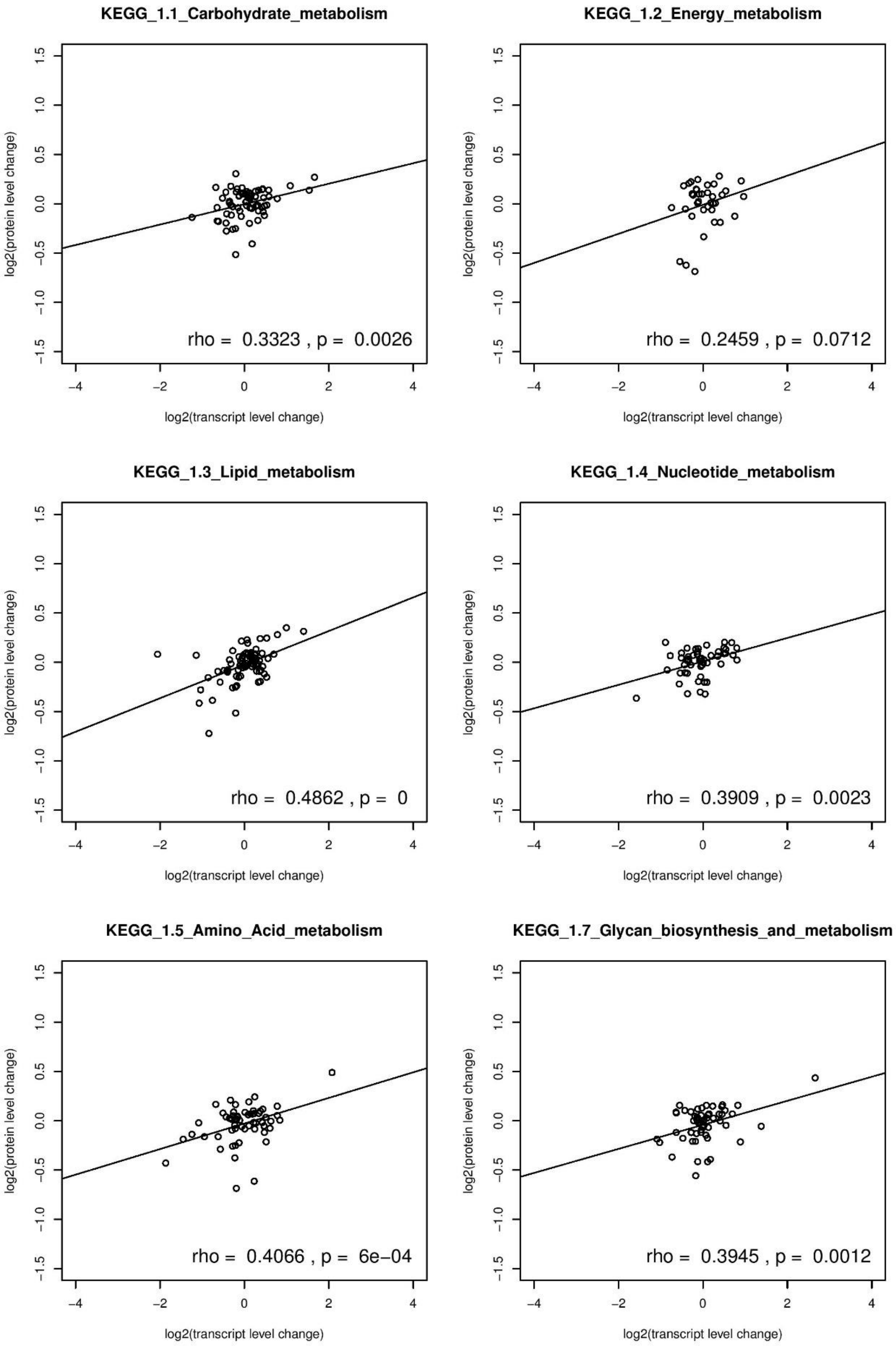

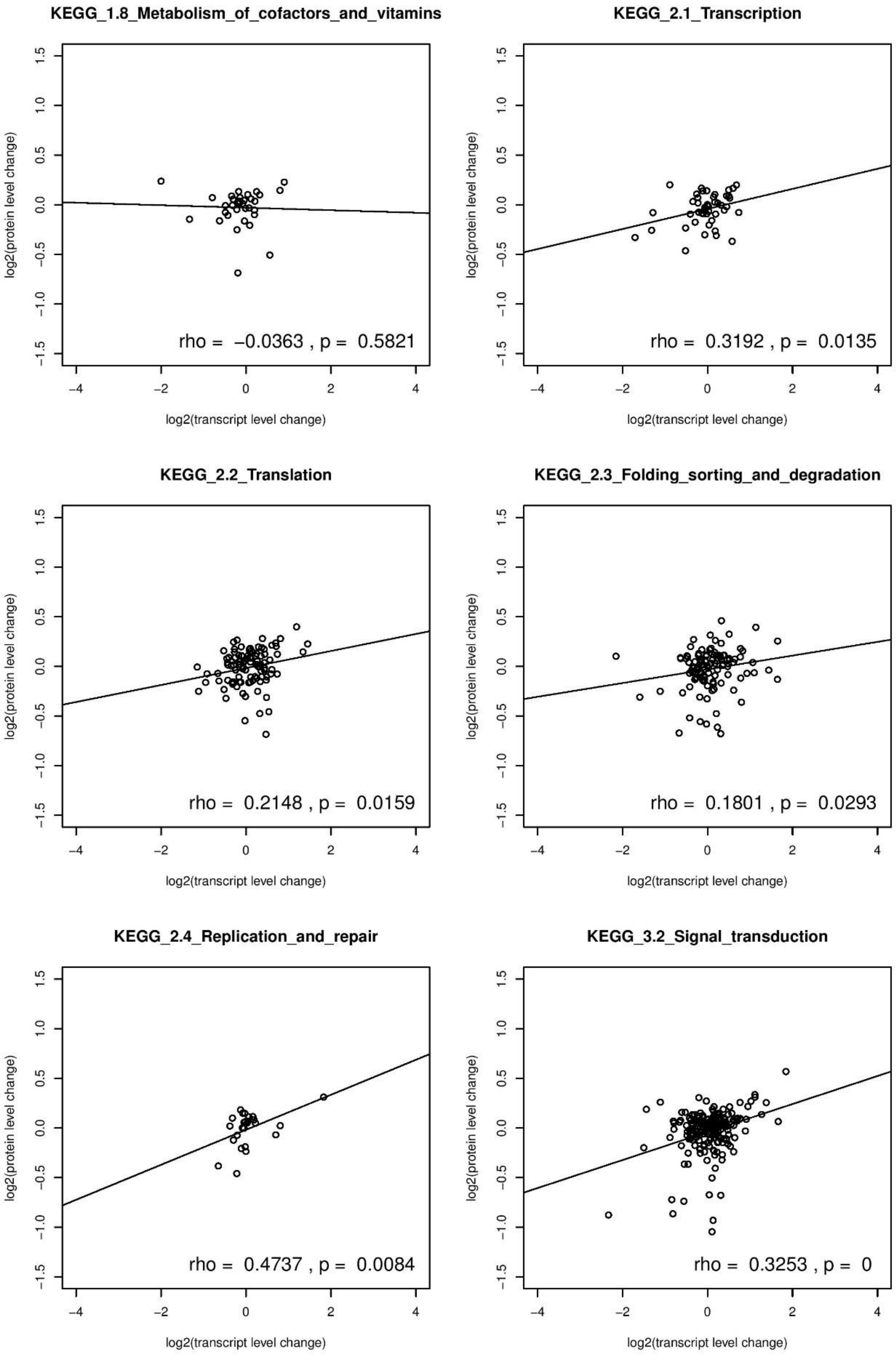

KEGG_1.8_Metabolism_of_cofactors_and_vitamins
rho = −0.0363 , p = 0.5821
KEGG_2.1_Transcription
rho = 0.3192 , p = 0.0135
KEGG_2.2_Translation
rho = 0.2148 , p = 0.0159
KEGG_2.3_Folding_sorting_and_degradation
rho = 0.1801 , p = 0.0293
KEGG_2.4_Replication_and_repair
rho = 0.4737 , p = 0.0084
KEGG_3.2_Signal_transduction
rho = 0.3253 , p = 0
log2(protein level change)
log2(transcript level change)
−1.5
−1.0
−0.5
0.0
0.5
1.0
1.5
−4
−2
0
2
4

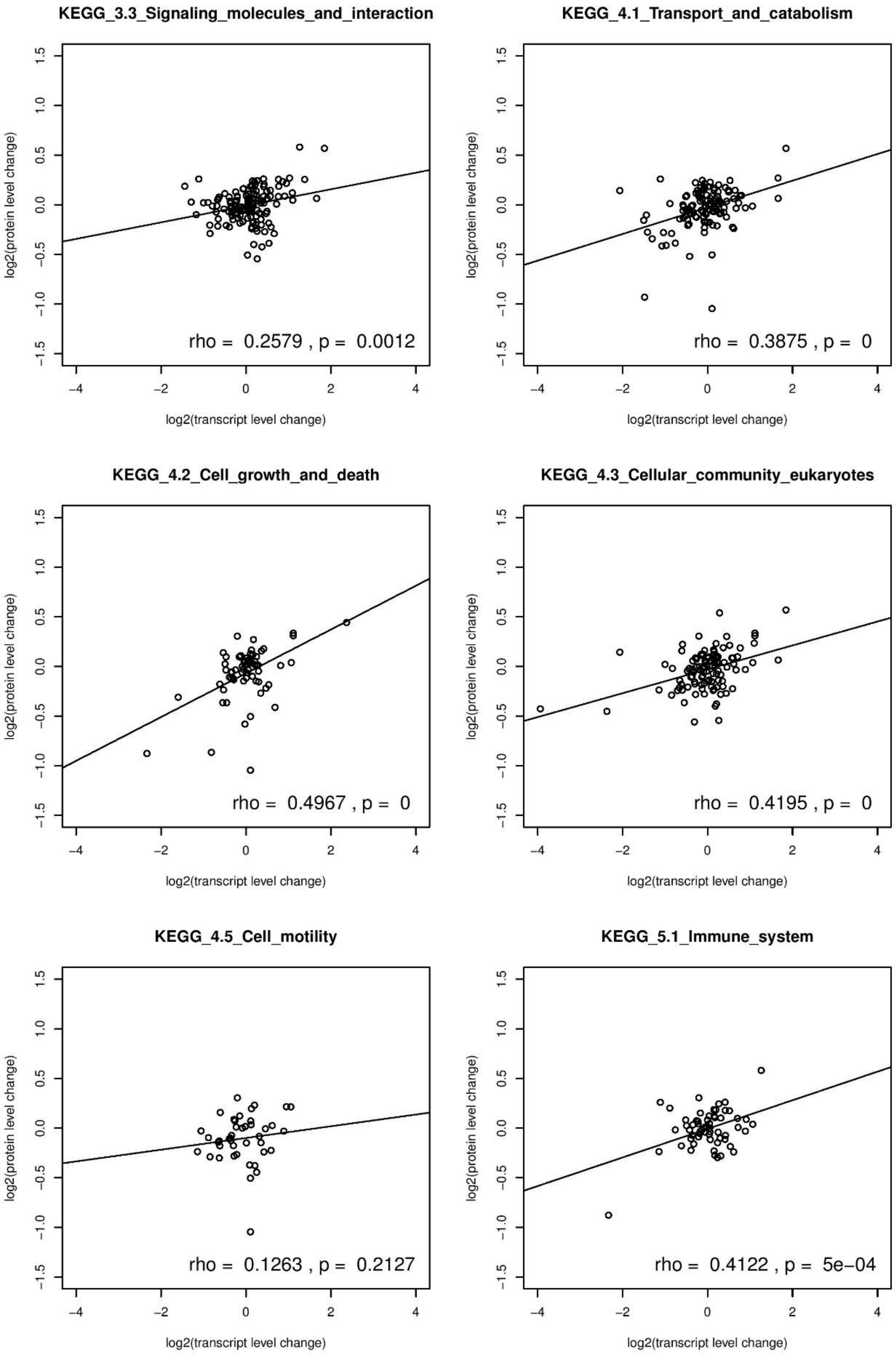
KEGG_3.3_Signaling_molecules_and_interaction
rho = 0.2579 , p = 0.0012
KEGG_4.1_Transport_and_catabolism
rho = 0.3875 , p = 0
KEGG_4.2_Cell_growth_and_death
rho = 0.4967 , p = 0
KEGG_4.3_Cellular_community_eukaryotes
rho = 0.4195 , p = 0
KEGG_4.5_Cell_motility
rho = 0.1263 , p = 0.2127
KEGG_5.1_Immune_system
rho = 0.4122 , p = 5e−04
log2(protein level change)
log2(transcript level change)
−1.5
−1.0
−0.5
0.0
0.5
1.0
1.5
−4
−2
0
2
4

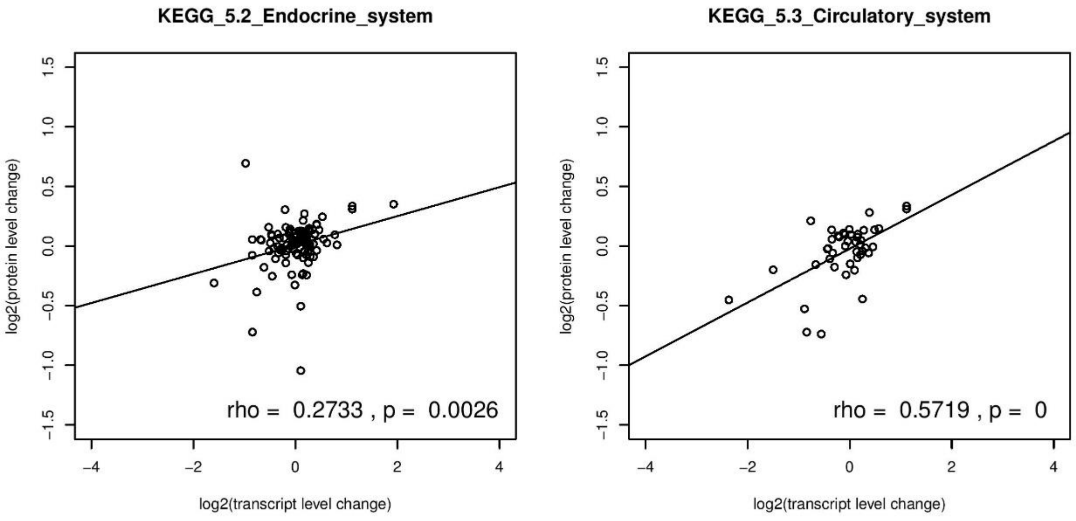

Figure S5. Correlation between transcript and protein level changes for KEGG pathway in AFB_1 sample. Only KEGG pathways with data size larger than 20 are plotted.

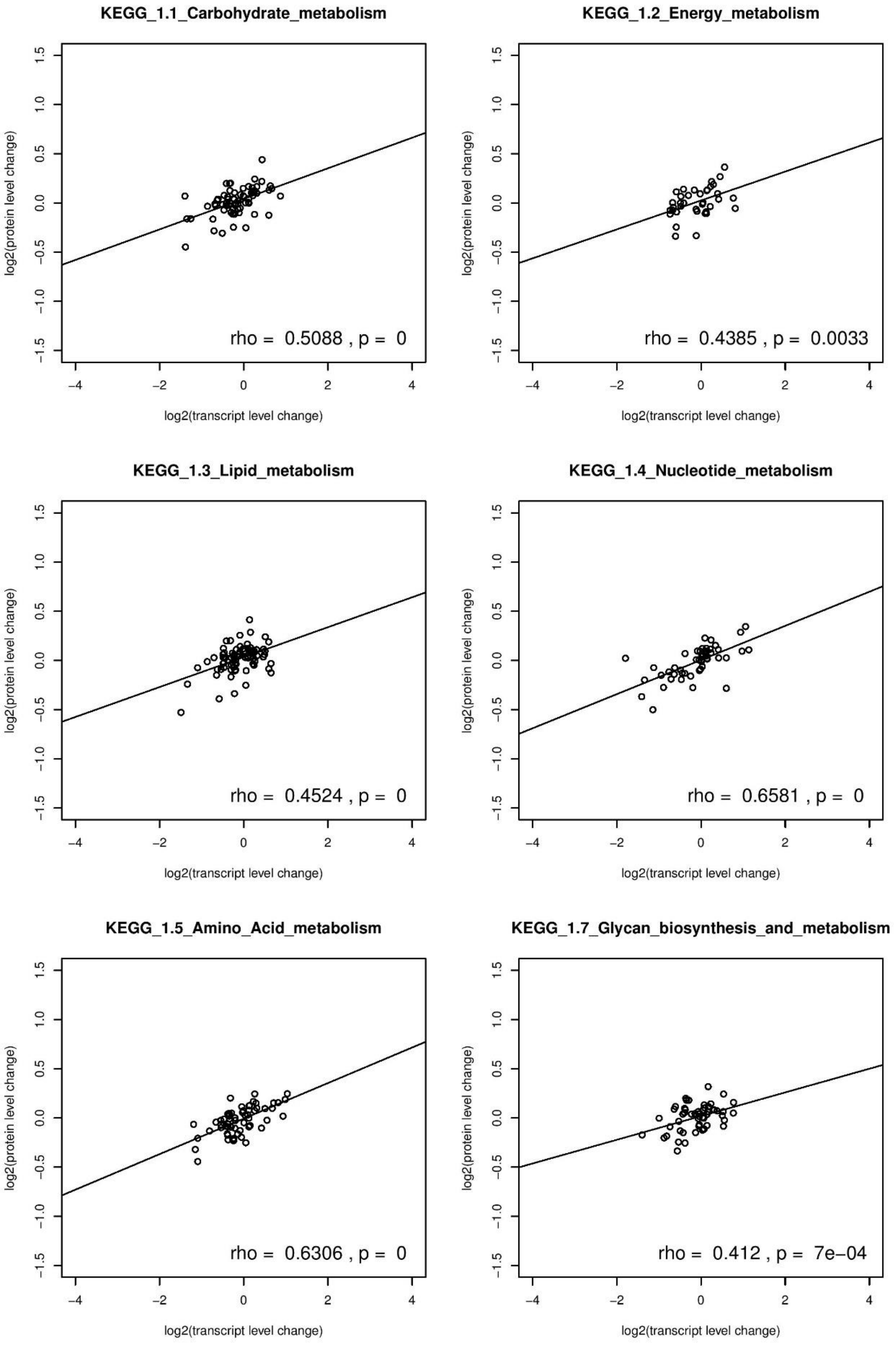
KEGG_1.1_Carbohydrate_metabolism
rho = 0.5088 , p = 0
KEGG_1.2_Energy_metabolism
rho = 0.4385 , p = 0.0033
KEGG_1.3_Lipid_metabolism
rho = 0.4524 , p = 0
KEGG_1.4_Nucleotide_metabolism
rho = 0.6581 , p = 0
KEGG_1.5_Amino_Acid_metabolism
rho = 0.6306 , p = 0
KEGG_1.7_Glycan_biosynthesis_and_metabolism
rho = 0.412 , p = 7e−04
log2(transcript level change)
log2(protein level change)

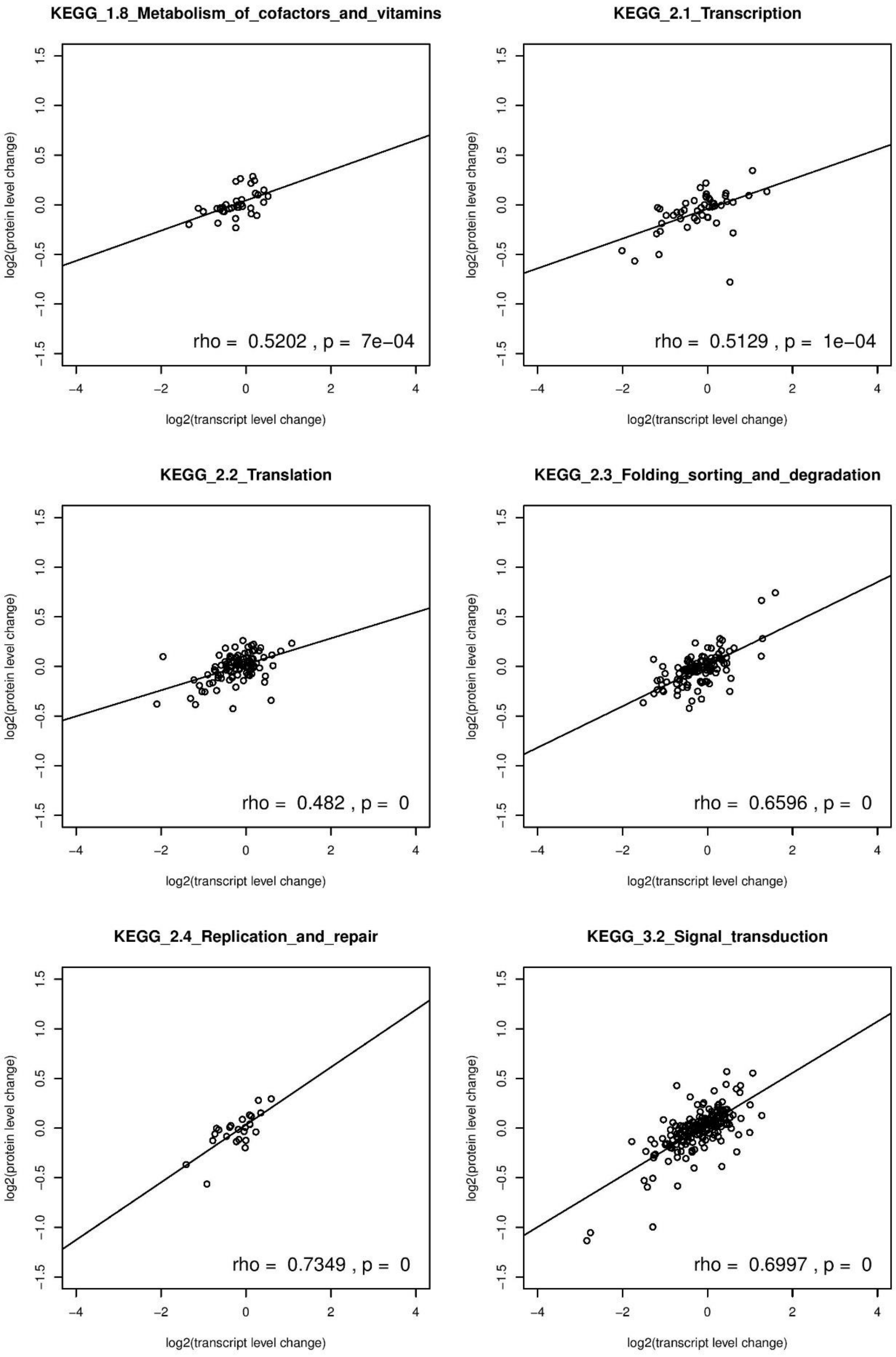
KEGG_1.8_Metabolism_of_cofactors_and_vitamins
rho = 0.5202 , p = 7e−04
KEGG_2.1_Transcription
rho = 0.5129 , p = 1e−04
KEGG_2.2_Translation
rho = 0.482 , p = 0
KEGG_2.3_Folding_sorting_and_degradation
rho = 0.6596 , p = 0
KEGG_2.4_Replication_and_repair
rho = 0.7349 , p = 0
KEGG_3.2_Signal_transduction
rho = 0.6997 , p = 0
log2(protein level change)
log2(transcript level change)
−1.5
−1.0
−0.5
0.0
0.5
1.0
1.5
−4
−2
0
2
4

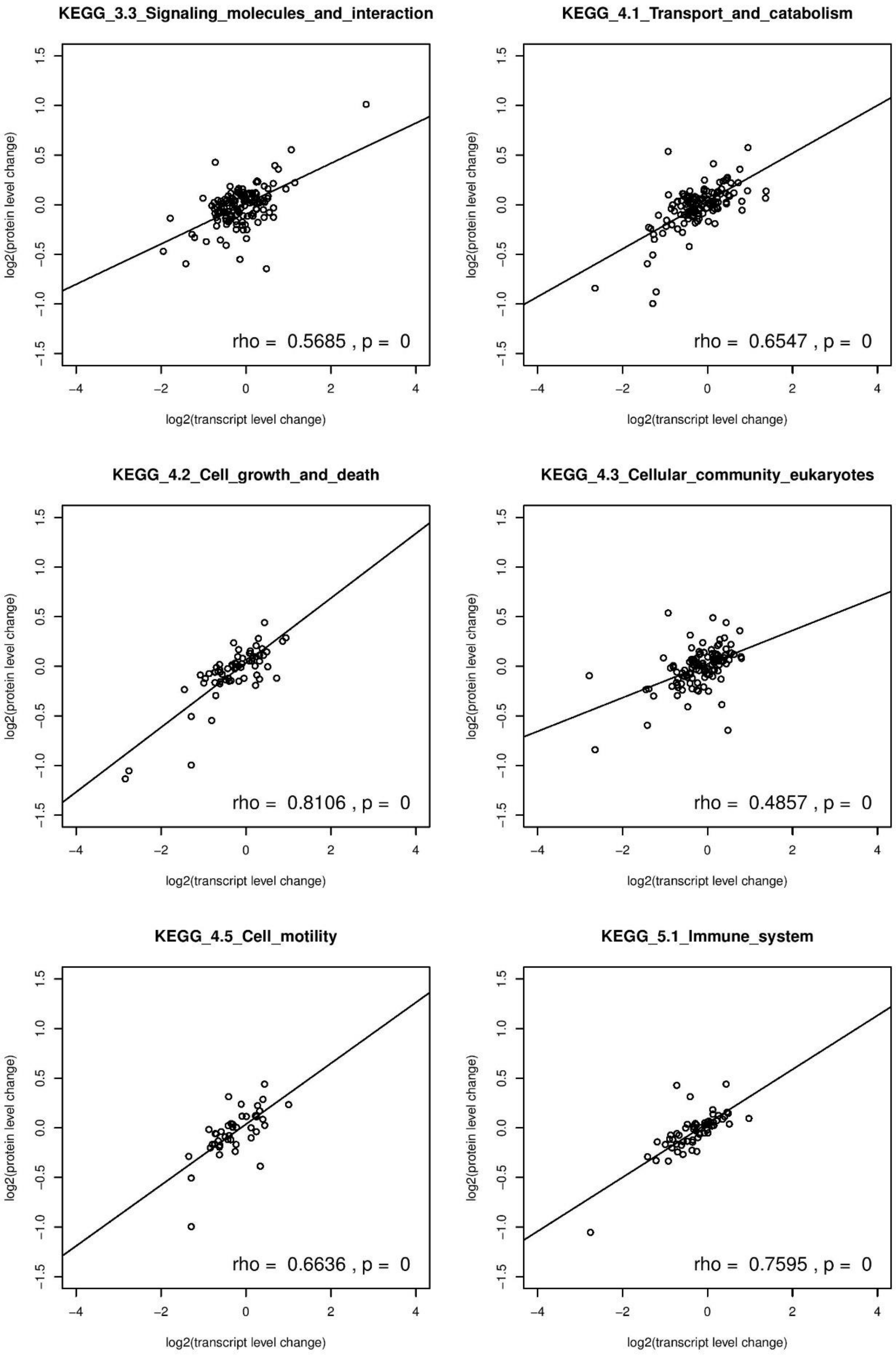
KEGG_3.3_Signaling_molecules_and_interaction
rho = 0.5685 , p = 0
KEGG_4.1_Transport_and_catabolism
rho = 0.6547 , p = 0
KEGG_4.2_Cell_growth_and_death
rho = 0.8106 , p = 0
KEGG_4.3_Cellular_community_eukaryotes
rho = 0.4857 , p = 0
KEGG_4.5_Cell_motility
rho = 0.6636 , p = 0
KEGG_5.1_Immune_system
rho = 0.7595 , p = 0
log2(protein level change)
log2(transcript level change)

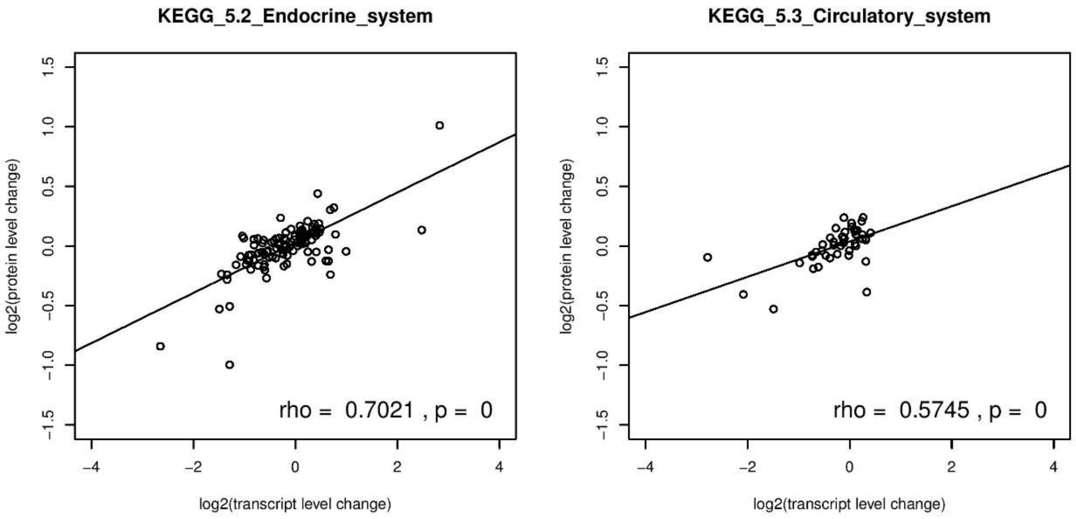

Figure S6. Correlation between transcript and protein level changes for KEGG pathway in OTA sample. Only KEGG pathways with data size larger than 20 are plotted.

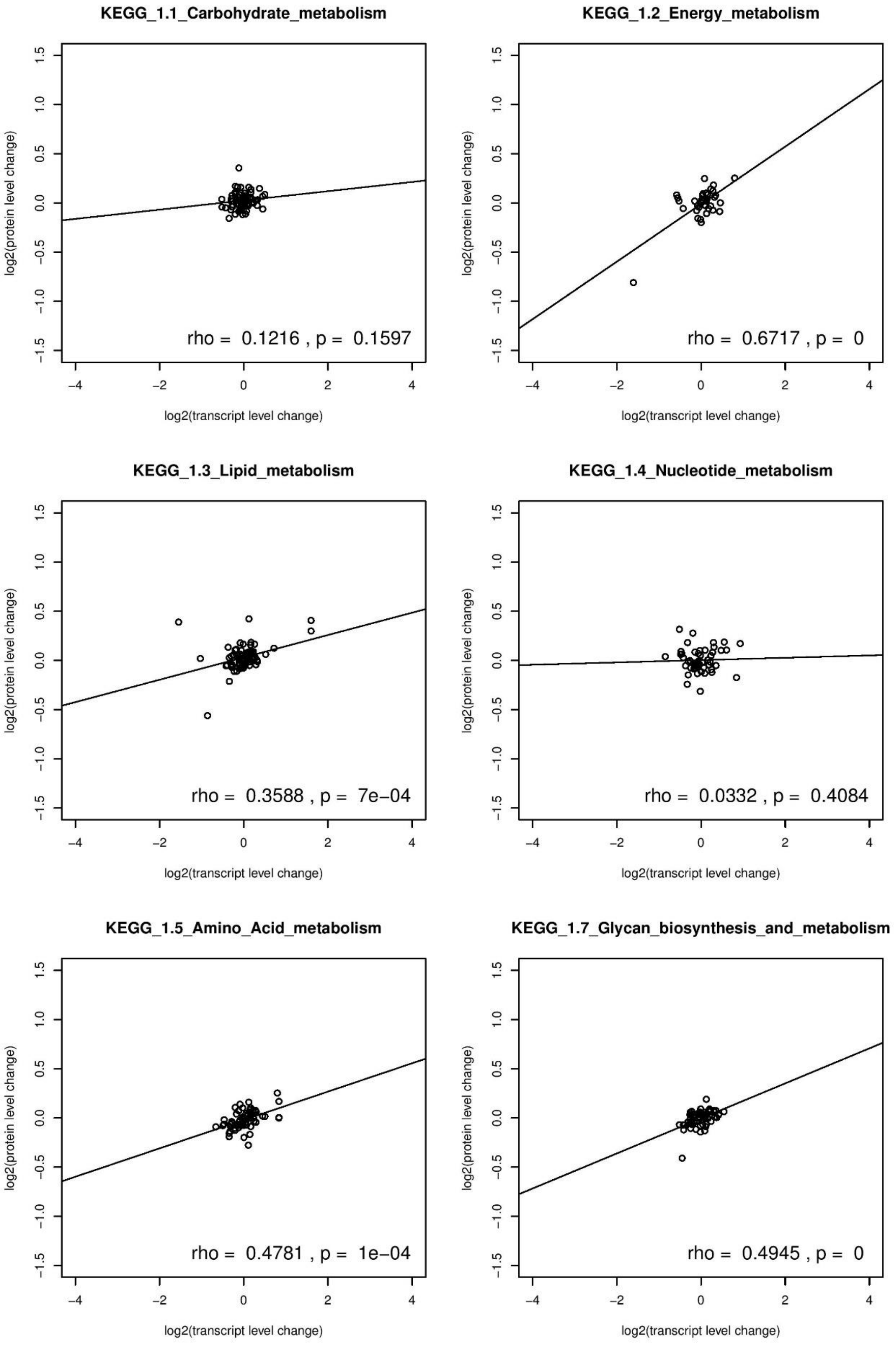
KEGG_1.1_Carbohydrate_metabolism
rho = 0.1216 , p = 0.1597
KEGG_1.2_Energy_metabolism
rho = 0.6717 , p = 0
KEGG_1.3_Lipid_metabolism
rho = 0.3588 , p = 7e−04
KEGG_1.4_Nucleotide_metabolism
rho = 0.0332 , p = 0.4084
KEGG_1.5_Amino_Acid_metabolism
rho = 0.4781 , p = 1e−04
KEGG_1.7_Glycan_biosynthesis_and_metabolism
rho = 0.4945 , p = 0
log2(protein level change)
log2(transcript level change)

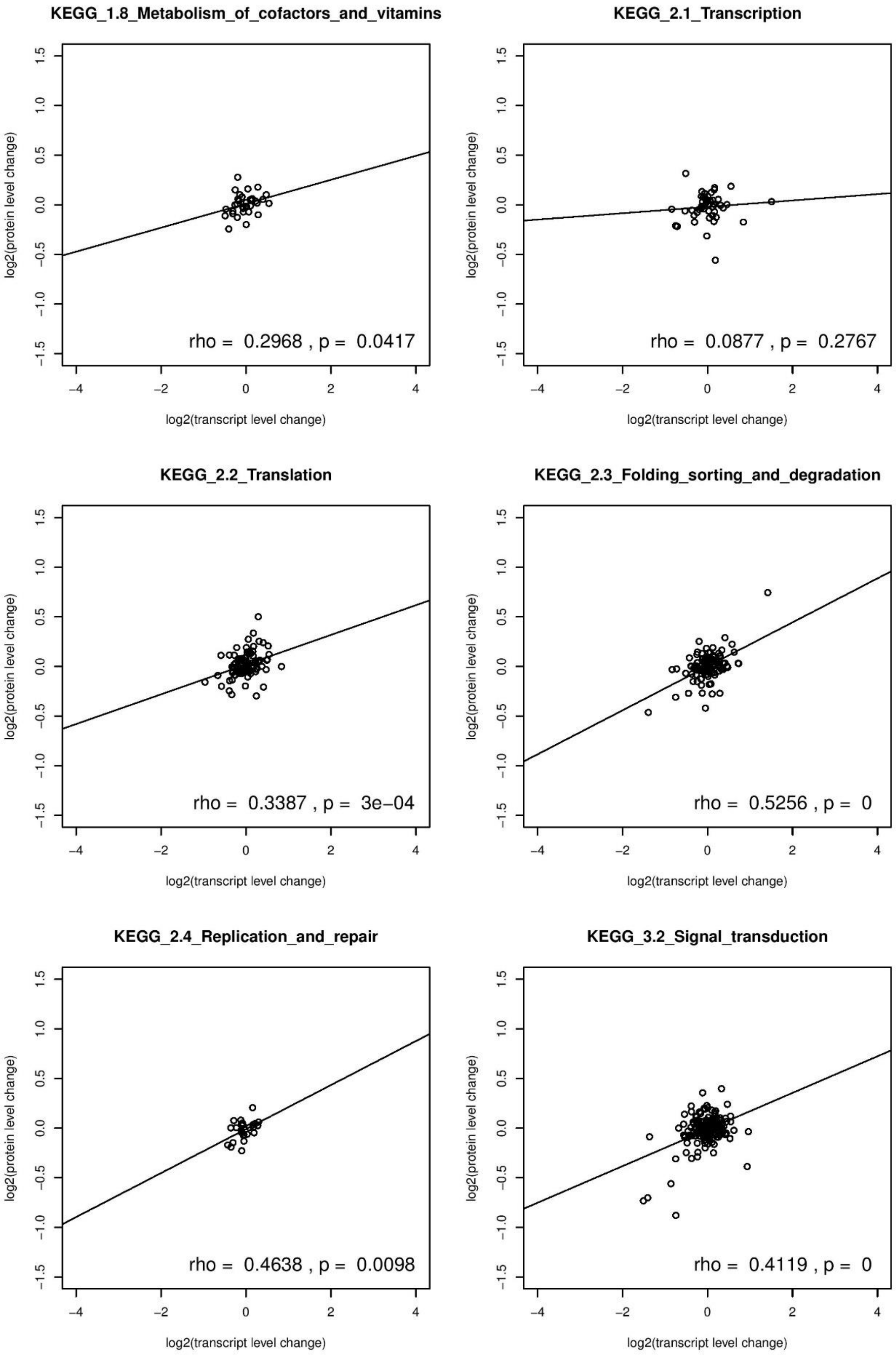
KEGG_1.8_Metabolism_of_cofactors_and_vitamins
rho = 0.2968 , p = 0.0417
KEGG_2.1_Transcription
rho = 0.0877 , p = 0.2767
KEGG_2.2_Translation
rho = 0.3387 , p = 3e−04
KEGG_2.3_Folding_sorting_and_degradation
rho = 0.5256 , p = 0
KEGG_2.4_Replication_and_repair
rho = 0.4638 , p = 0.0098
KEGG_3.2_Signal_transduction
rho = 0.4119 , p = 0
log2(protein level change)
log2(transcript level change)
−1.5
−1.0
−0.5
0.0
0.5
1.0
1.5
−4
−2
0
2
4

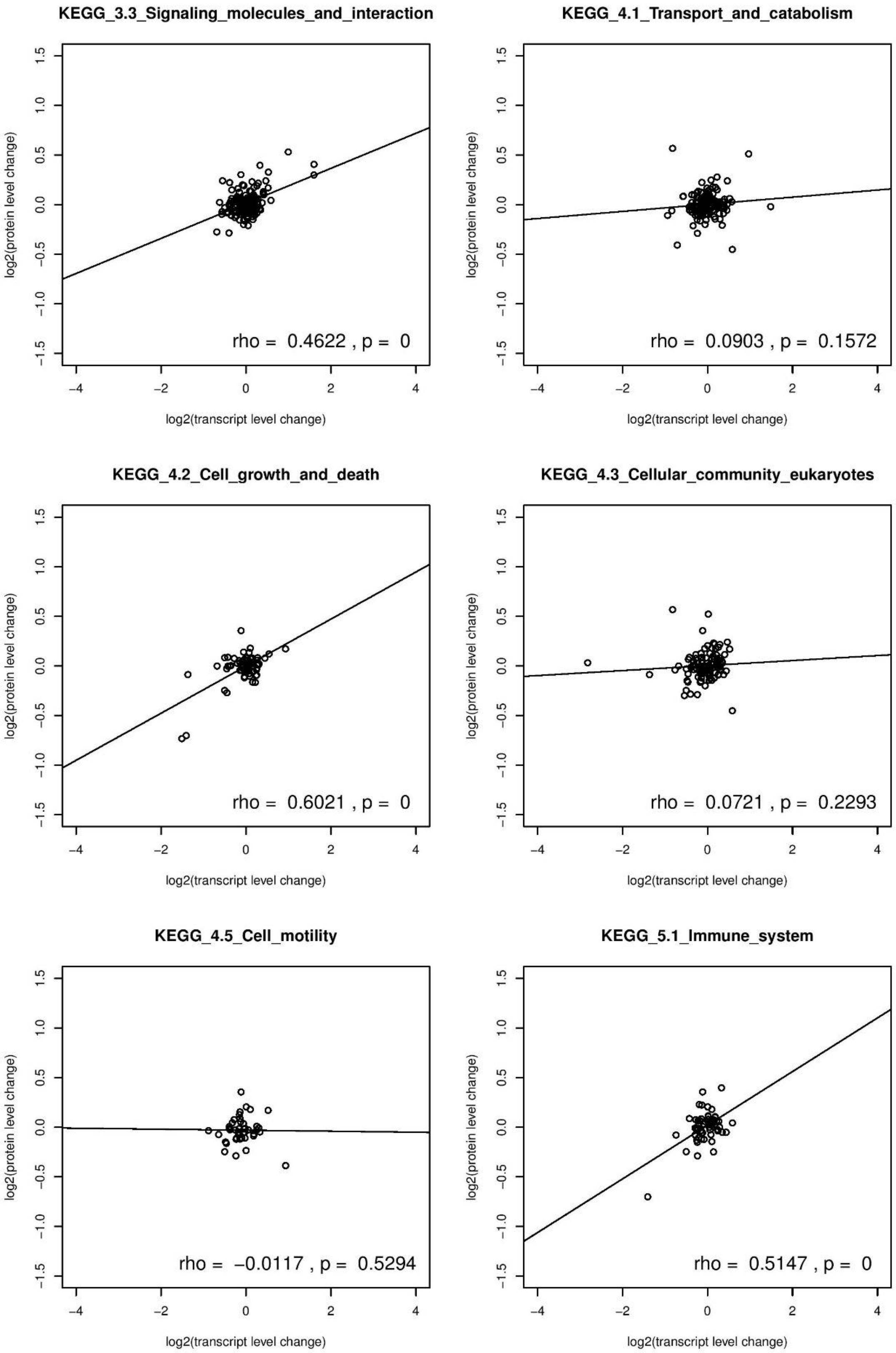
KEGG_3.3_Signaling_molecules_and_interaction
rho = 0.4622 , p = 0
KEGG_4.1_Transport_and_catabolism
rho = 0.0903 , p = 0.1572
KEGG_4.2_Cell_growth_and_death
rho = 0.6021 , p = 0
KEGG_4.3_Cellular_community_eukaryotes
rho = 0.0721 , p = 0.2293
KEGG_4.5_Cell_motility
rho = −0.0117 , p = 0.5294
KEGG_5.1_Immune_system
rho = 0.5147 , p = 0
log2(protein level change)
log2(transcript level change)
−1.5
−1.0
−0.5
0.0
0.5
1.0
1.5
−4
−2
0
2
4

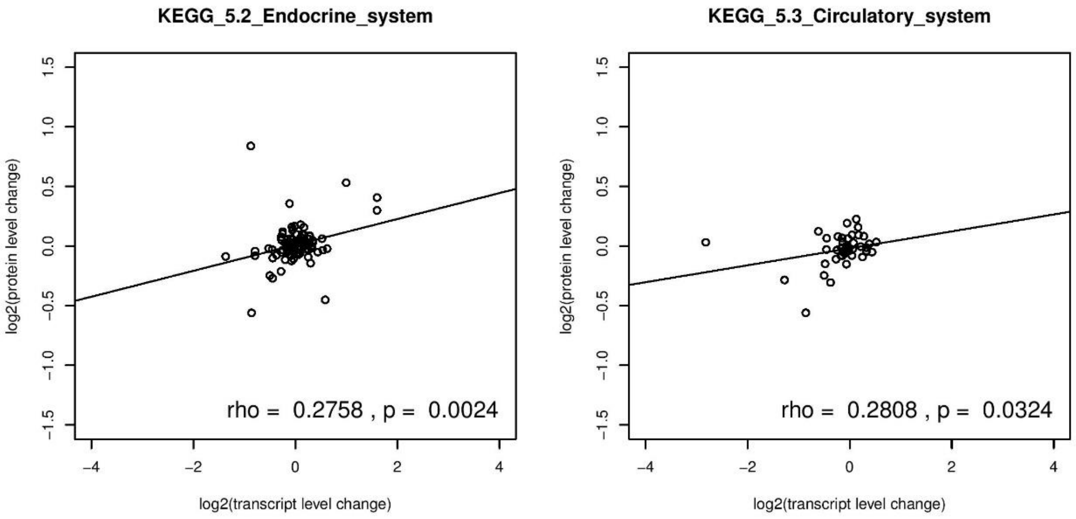

Figure S7. Correlation between transcript and protein level changes for KEGG pathway in ZEN sample. Only KEGG pathways with data size larger than 20 are plotted.

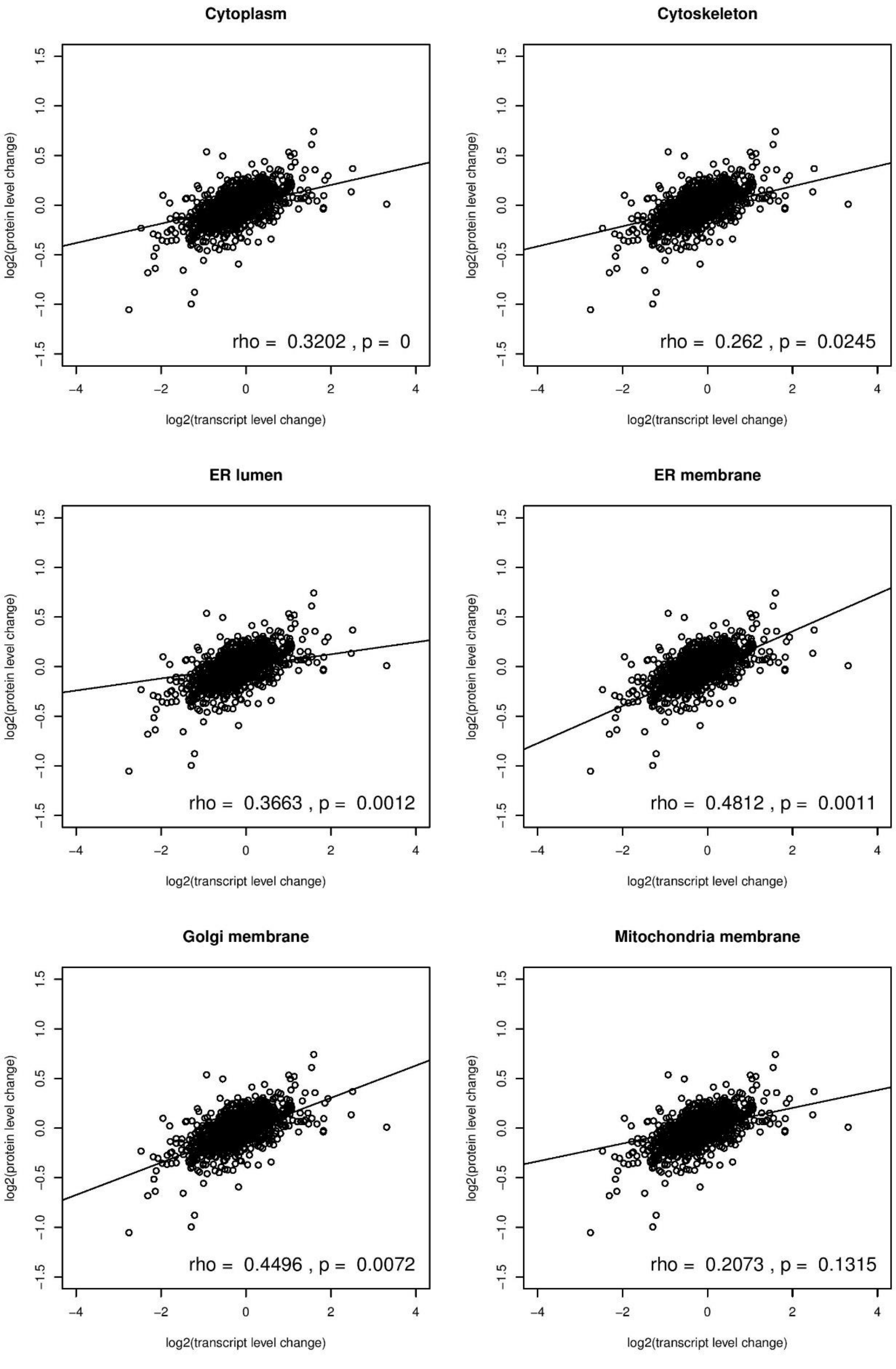
Cytoplasm
rho = 0.3202 , p = 0
Cytoskeleton
rho = 0.262 , p = 0.0245
ER lumen
rho = 0.3663 , p = 0.0012
ER membrane
rho = 0.4812 , p = 0.0011
Golgi membrane
rho = 0.4496 , p = 0.0072
Mitochondria membrane
rho = 0.2073 , p = 0.1315
log2(protein level change)
log2(transcript level change)
−1.5
−1.0
−0.5
0.0
0.5
1.0
1.5
−4
−2
0
2
4

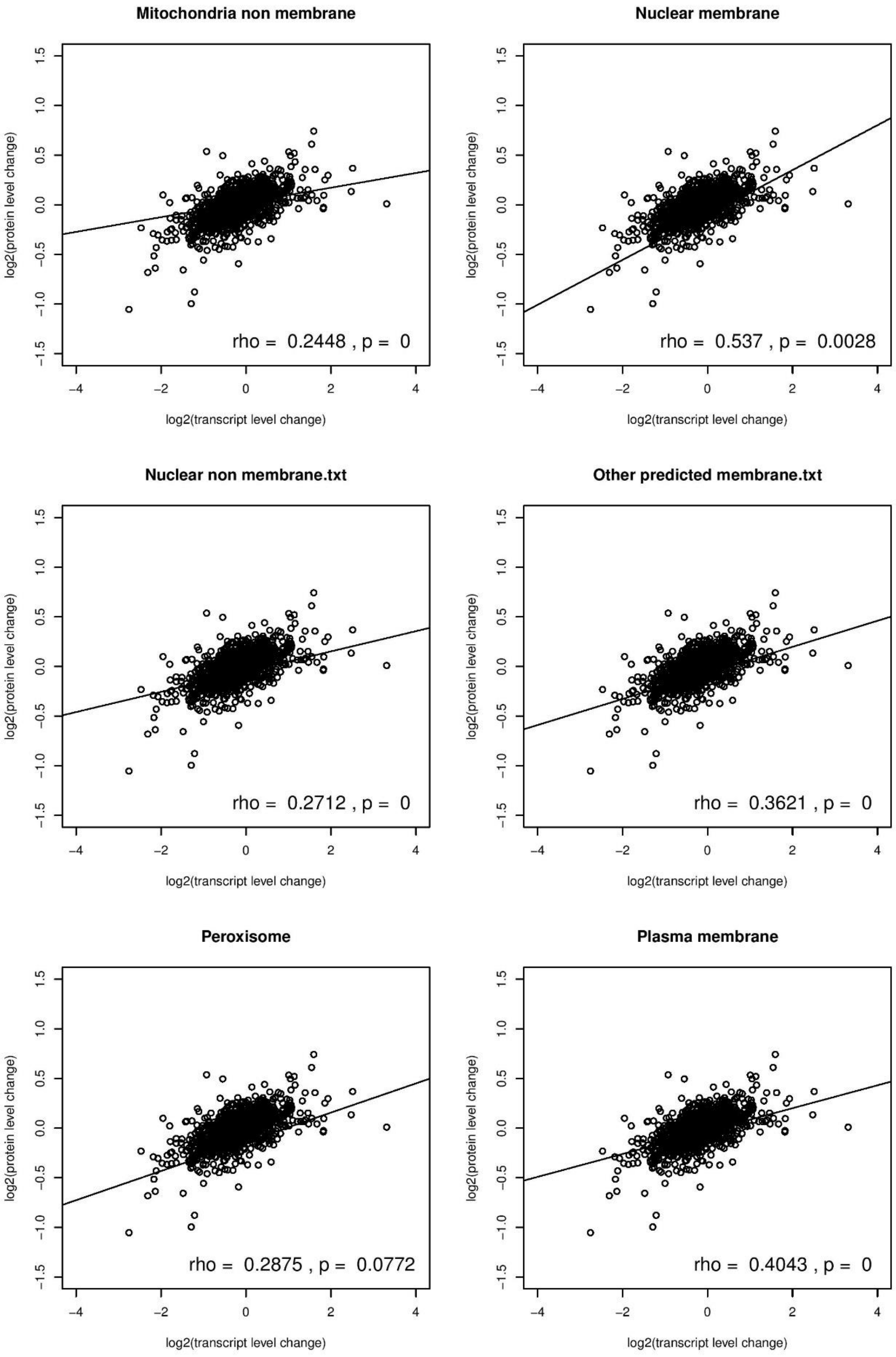

Mitochondria non membrane
rho = 0.2448 , p = 0
Nuclear membrane
rho = 0.537 , p = 0.0028
Nuclear non membrane.txt
rho = 0.2712 , p = 0
Other predicted membrane.txt
rho = 0.3621 , p = 0
Peroxisome
rho = 0.2875 , p = 0.0772
Plasma membrane
rho = 0.4043 , p = 0
log2(protein level change)
log2(transcript level change)

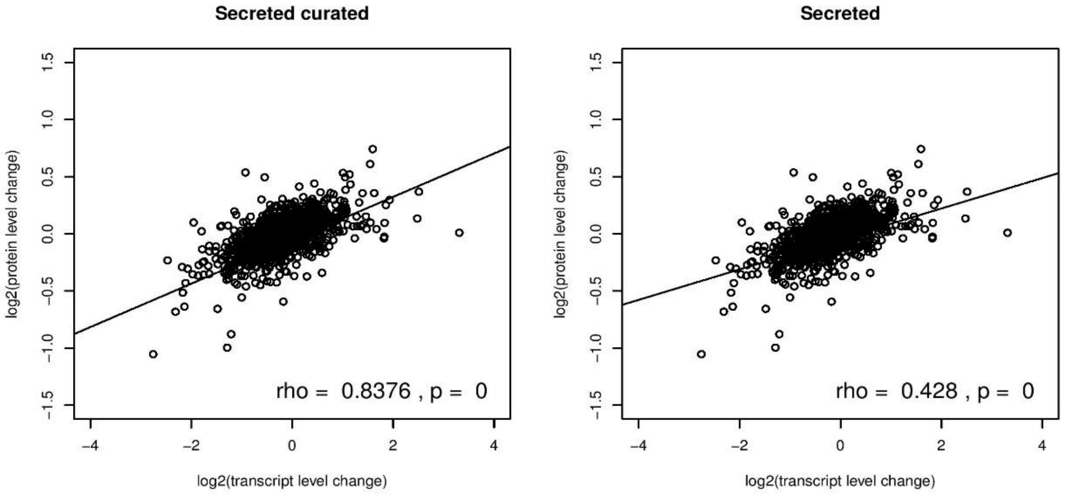

Figure S8. Correlation between transcript and protein level changes for protein subcellular location in AFB_1 sample. Only subcellular location with data size larger than 10 are plotted.

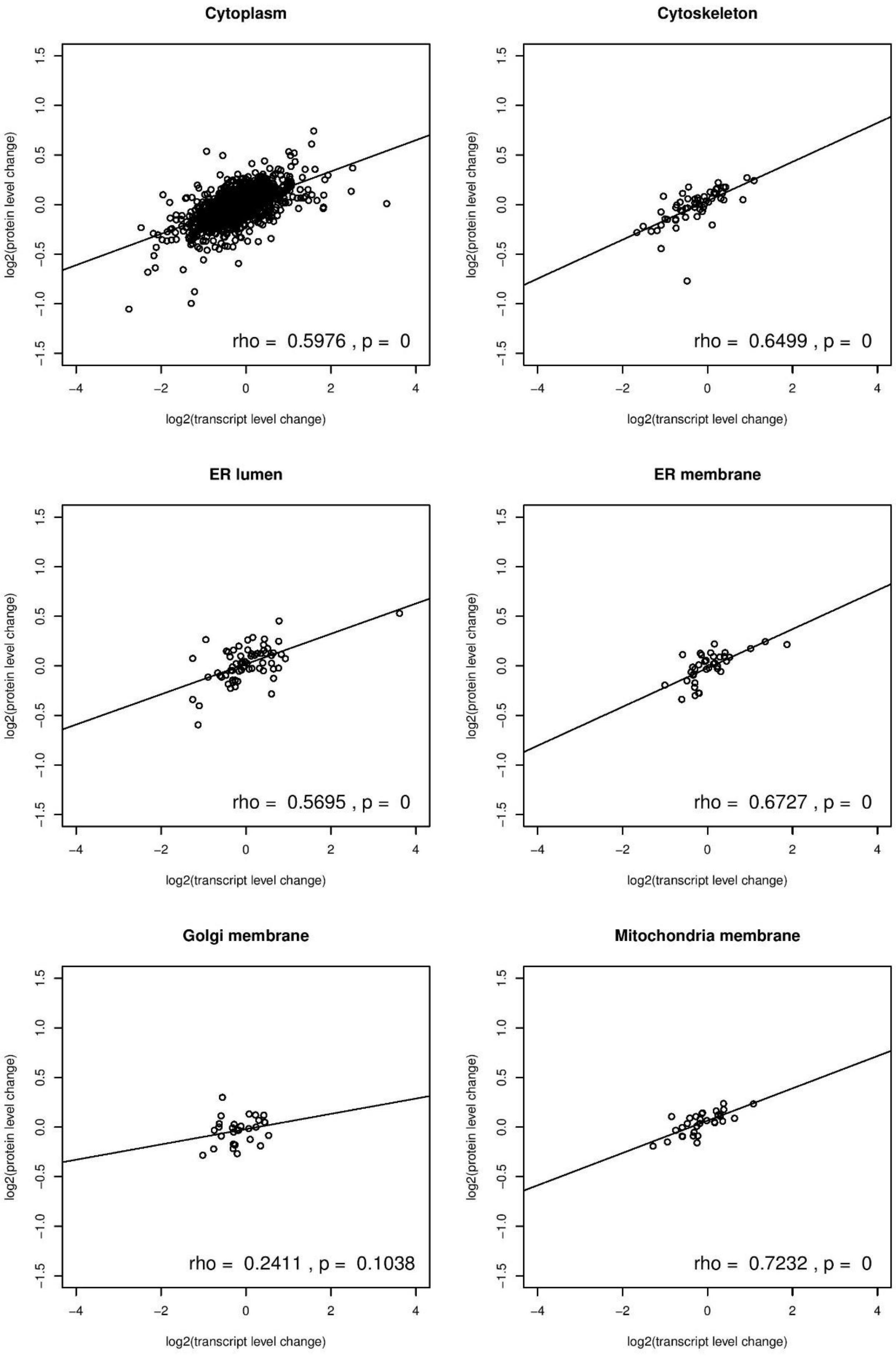
Cytoplasm
rho = 0.5976 , p = 0
Cytoskeleton
rho = 0.6499 , p = 0
ER lumen
rho = 0.5695 , p = 0
ER membrane
rho = 0.6727 , p = 0
Golgi membrane
rho = 0.2411 , p = 0.1038
Mitochondria membrane
rho = 0.7232 , p = 0
log2(protein level change)
log2(transcript level change)

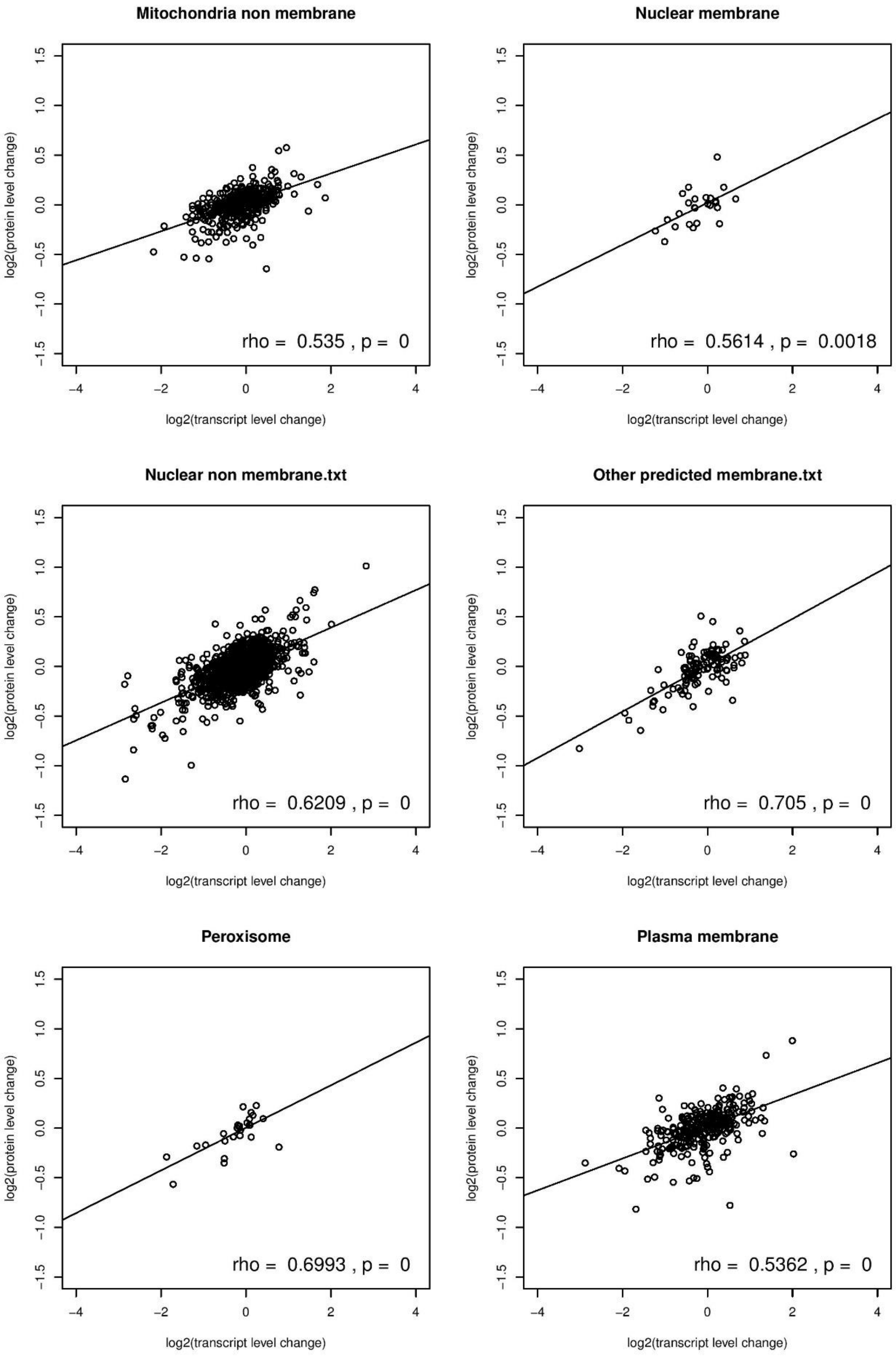

Mitochondria non membrane
rho = 0.535 , p = 0
Nuclear membrane
rho = 0.5614 , p = 0.0018
Nuclear non membrane.txt
rho = 0.6209 , p = 0
Other predicted membrane.txt
rho = 0.705 , p = 0
Peroxisome
rho = 0.6993 , p = 0
Plasma membrane
rho = 0.5362 , p = 0
log2(protein level change)
log2(transcript level change)

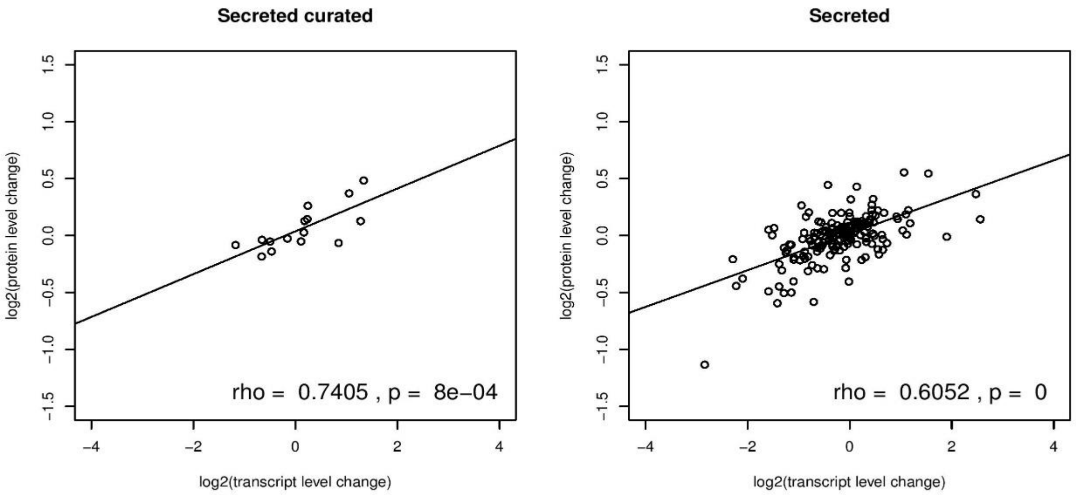

Figure S9. Correlation between transcript and protein level changes for protein subcellular location in OTA sample. Only subcellular location with data size larger than 10 are plotted.

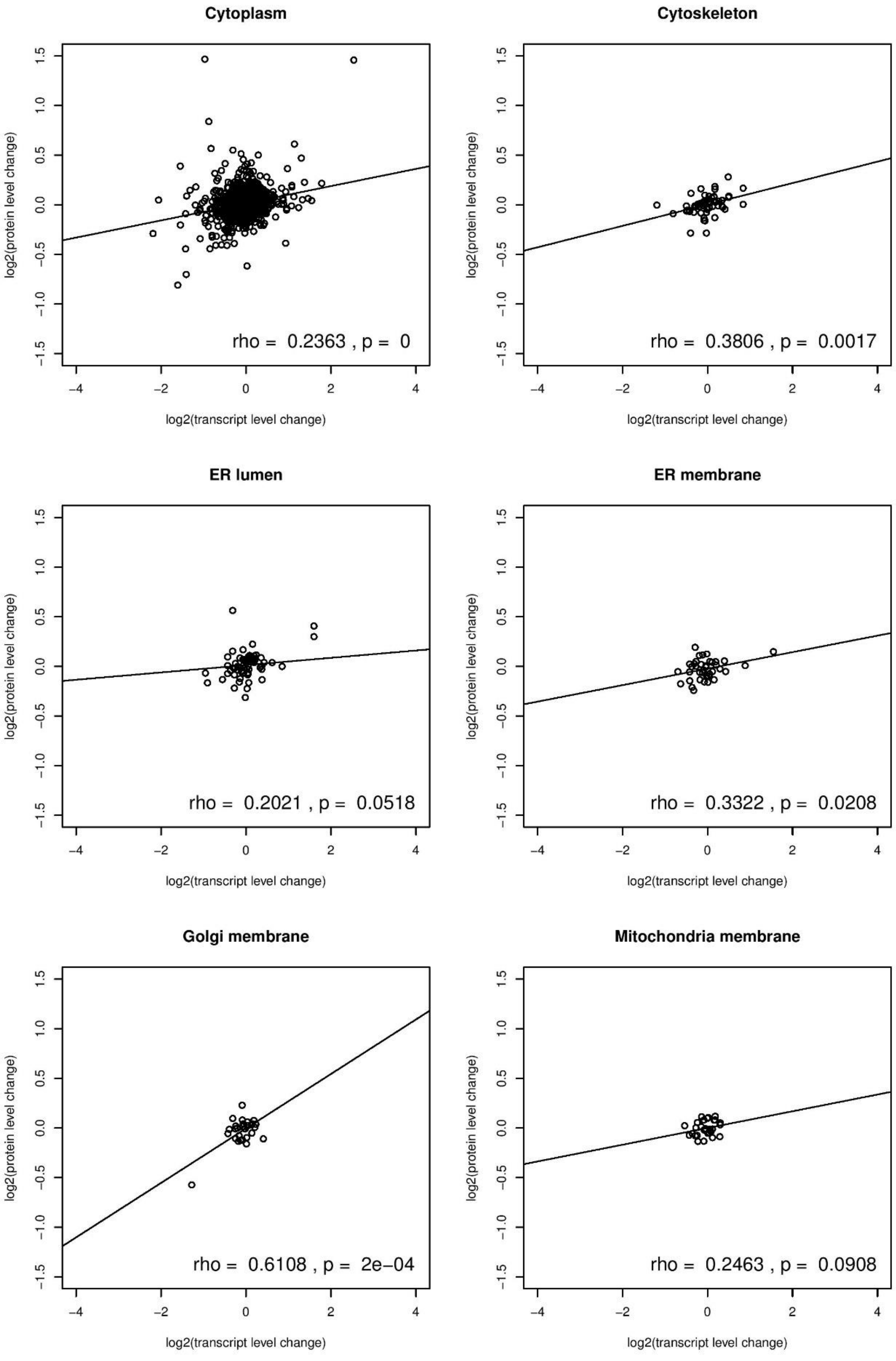
Cytoplasm
rho = 0.2363 , p = 0
Cytoskeleton
rho = 0.3806 , p = 0.0017
ER lumen
rho = 0.2021 , p = 0.0518
ER membrane
rho = 0.3322 , p = 0.0208
Golgi membrane
rho = 0.6108 , p = 2e−04
Mitochondria membrane
rho = 0.2463 , p = 0.0908
log2(protein level change)
log2(transcript level change)
−1.5
−1.0
−0.5
0.0
0.5
1.0
1.5
−4
−2
0
2
4

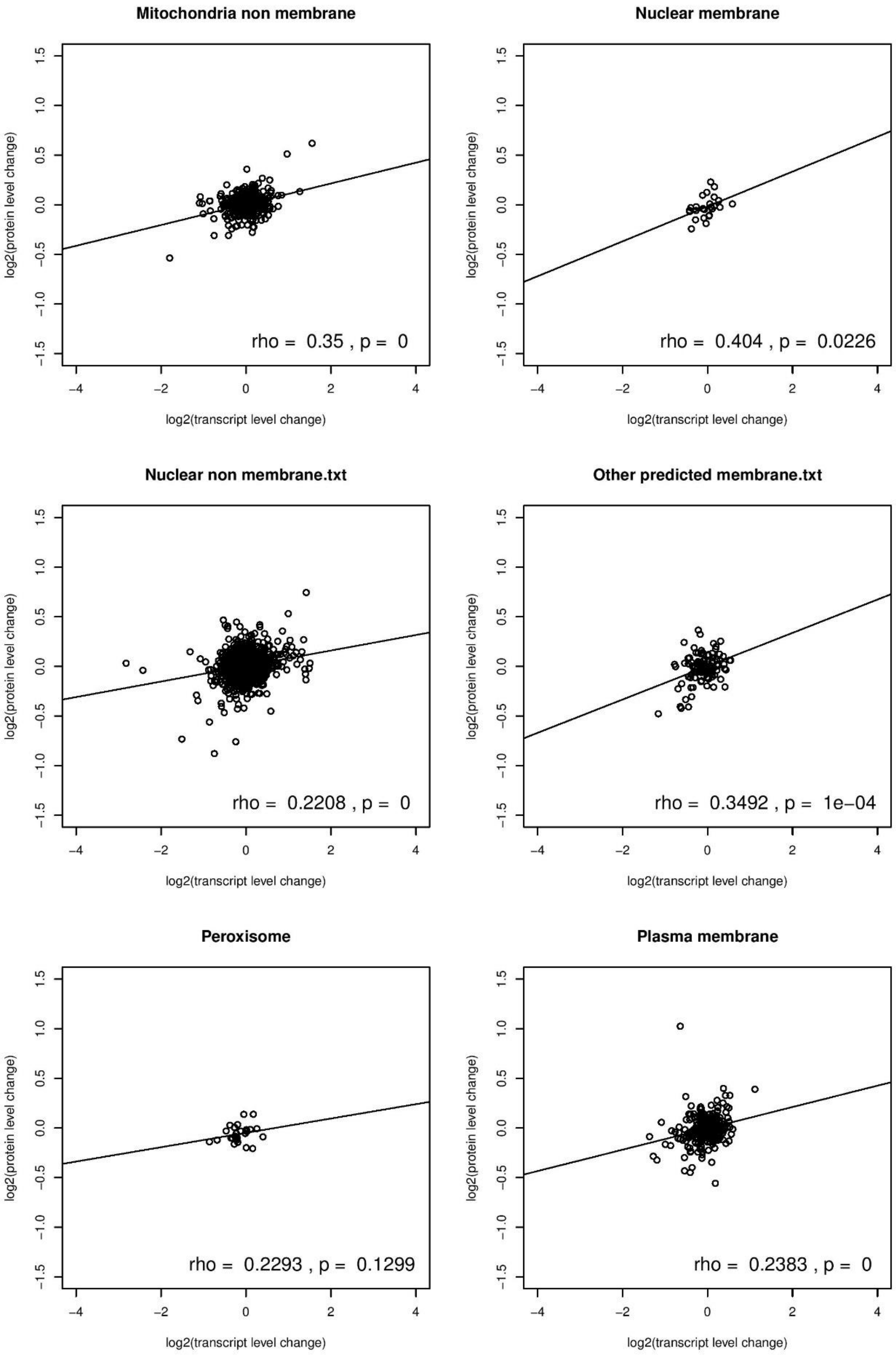
Mitochondria non membrane
rho = 0.35 , p = 0
Nuclear membrane
rho = 0.404 , p = 0.0226
Nuclear non membrane.txt
rho = 0.2208 , p = 0
Other predicted membrane.txt
rho = 0.3492 , p = 1e−04
Peroxisome
rho = 0.2293 , p = 0.1299
Plasma membrane
rho = 0.2383 , p = 0
log2(protein level change)
log2(transcript level change)
−1.5
−1.0
−0.5
0.0
0.5
1.0
1.5
−4
−2
0
2
4

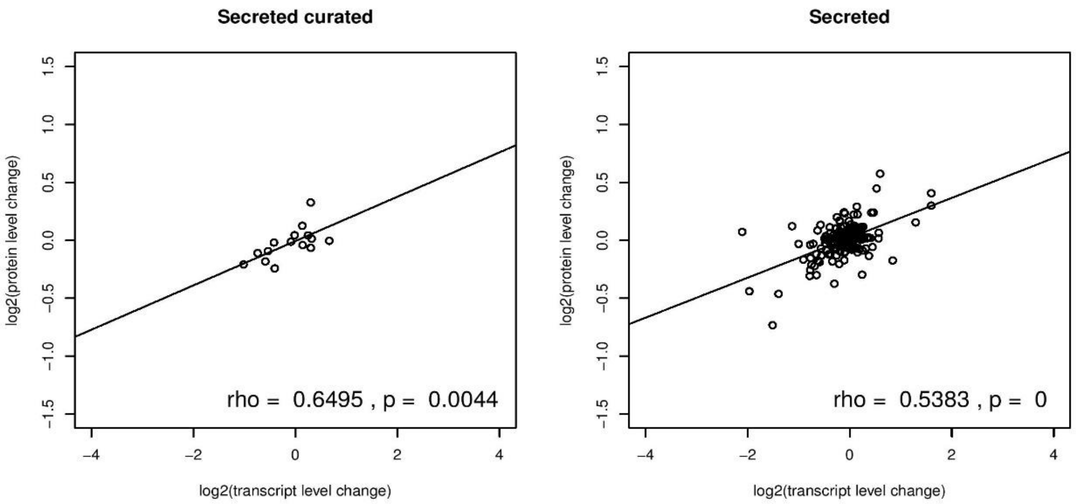

Figure S10. Correlation between transcript and protein level changes for protein subcellular location in ZEN sample. Only subcellular location with data size larger than 10 are plotted.

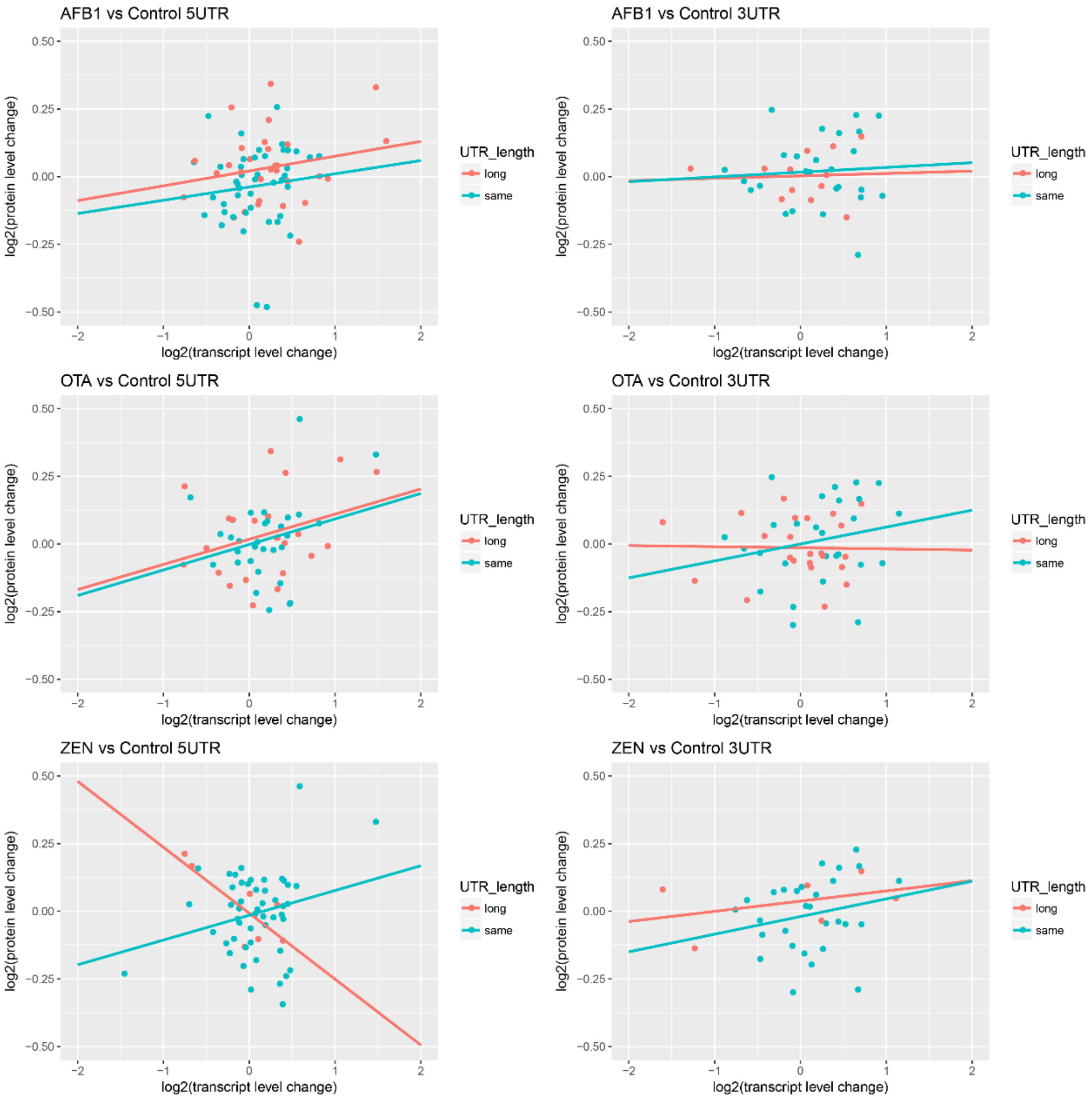

Figure S11. Transcript and protein level changes for long UTR and UTR of same length.

Supplementary tables:

TableS1. Transcriptome data statistics for all sequenced samples.

**Sample name**	**Raw reads**	**Clean reads**	**clean bases**	**Error rate(%)**	**Q20(%)**	**Q30(%)**	**GC content(%)**
Control_1	48011464	46146592	6.92G	0.02	96.29	91.39	49.84
Control_2	53128276	50112460	7.52G	0.02	95.47	89.89	51.43
Control_3	44275030	42295782	6.34G	0.02	96.06	90.92	50.8
AFB1_1	48520062	46521682	6.98G	0.02	96.42	91.67	52.53
AFB1_2	51395982	49121320	7.37G	0.02	96.13	91.11	51.41
AFB1_3	42786932	40780346	6.12G	0.02	95.95	90.76	50.66
OTA_1	47164368	45247736	6.79G	0.02	96.34	91.51	50.39
OTA_2	48277552	45939754	6.89G	0.02	95.87	90.62	51.14
OTA_3	44776390	42798438	6.42G	0.02	96.13	91.09	50.94
ZEN_1	52354878	50143446	7.52G	0.02	96.17	91.18	50.86
ZEN_2	46068220	43970064	6.6G	0.02	95.95	90.76	50.92
ZEN_3	50849228	48784196	7.32G	0.02	96.24	91.3	51.34

TableS2. Proteome data statistics for all sequenced samples.

**Database**	**Replicate**	**Total spectra**	**Spectra (PSM)**	**Peptides**	**Unique peptides**	**Protein groups**
Chicken	1	228477	82747	46714	42353	6608
Chicken	2	288995	86688	47680	43348	6619
Chicken	3	288263	83880	47402	43170	6645
Chicken	1,2,3 merged	865735	253315	68759	61689	7491

Table S3. The number of differentially expressed genes in three mycotoxin.

**Toxin**	**RNA UP**	**RNA DOWN**	**Total DEG**	**Prot UP**	**Prot DOWN**	**Total DEG**
**AFB1**	3583	3411	6994	190	466	656
**OTA**	4496	4161	8657	201	347	548
**ZEN**	1692	1856	3548	108	108	216

TableS4 Correlation between transcript and protein level changes for KEGG pathway.

**kegg pathway**	**rho_afb1**	**pvalue_afb1**	**slope_afb1**	**rho_ota**	**pvalue_ota**	**slope_ota**	**rho_zen**	**pvalue_zen**	**slope_zen**	**data_size_zen**
kegg_1.1	0.3323	2.64E-03	0.1037	0.509	4.02E-06	0.155	0.1216	1.60E-01	0.04693	69
kegg_1.2	0.2459	7.12E-02	0.1475	0.438	3.32E-03	0.147	0.6717	2.66E-06	0.29251	37
kegg_1.3	0.4862	3.69E-06	0.1705	0.452	1.81E-05	0.152	0.3588	6.78E-04	0.11367	77
kegg_1.4	0.3909	2.28E-03	0.1191	0.658	7.70E-08	0.174	0.0332	4.08E-01	0.01182	51
kegg_1.5	0.4066	6.32E-04	0.1298	0.631	3.33E-08	0.181	0.4781	5.59E-05	0.14428	60
kegg_1.6	0.3427	8.91E-02	0.1096	0.394	5.87E-02	0.123	0.4715	2.80E-02	0.17873	17
kegg_1.7	0.3945	1.20E-03	0.122	0.412	7.26E-04	0.12	0.4945	4.62E-05	0.17816	57
kegg_1.8	-0.0363	5.82E-01	-0.0126	0.52	6.80E-04	0.152	0.2968	4.17E-02	0.12076	35
kegg_1.9	NA	NA	NA	NA	NA	NA	NA	NA	NA	4
kegg_1.10	NA	NA	NA	NA	NA	NA	NA	NA	NA	2
kegg_1.11	0.5344	1.36E-02	0.162	0.454	3.37E-02	0.116	-0.1846	7.61E-01	-0.08198	17
kegg_1.12	NA	NA	NA	NA	NA	NA	NA	NA	NA	0
kegg_2.1	0.3192	1.35E-02	0.1011	0.513	9.67E-05	0.15	0.0877	2.77E-01	0.03209	48
kegg_2.2	0.2148	1.59E-02	0.0857	0.482	1.91E-07	0.131	0.3387	2.83E-04	0.14977	100
kegg_2.3	0.1801	2.93E-02	0.0692	0.66	1.73E-15	0.209	0.5256	1.58E-09	0.22164	111

kegg_2.4	0.4737	8.37E-03	0.1764	0.735	1.43E-05	0.29	0.4638	9.76E-03	0.22184	25
kegg_3.1	0.926	2.15E-05	0.2206	0.898	8.61E-05	0.468	0.3044	1.81E-01	0.14821	11
kegg_3.2	0.3253	2.94E-06	0.1411	0.7	5.67E-29	0.259	0.4119	2.60E-09	0.18453	186
kegg_3.3	0.2579	1.17E-03	0.0834	0.568	2.14E-13	0.203	0.4622	6.50E-09	0.17684	137
kegg_4.1	0.3875	3.67E-06	0.1344	0.655	4.57E-17	0.241	0.0903	1.57E-01	0.03593	126
kegg_4.2	0.4967	1.29E-05	0.2207	0.811	1.40E-16	0.326	0.6021	5.62E-08	0.23752	65
kegg_4.3	0.4195	3.10E-06	0.1204	0.486	4.95E-08	0.169	0.0721	2.29E-01	0.02525	108
kegg_4.4	NA	NA	NA	NA	NA	NA	NA	NA	NA	0
kegg_4.5	0.1263	2.13E-01	0.0591	0.664	8.32E-07	0.307	-0.0117	5.29E-01	-0.00505	42
kegg_5.1	0.4122	5.33E-04	0.1441	0.759	1.00E-12	0.272	0.5147	1.29E-05	0.27059	60
kegg_5.2	0.2733	2.61E-03	0.1215	0.702	7.07E-17	0.211	0.2758	2.40E-03	0.10874	103
kegg_5.3	0.5719	2.51E-05	0.2257	0.575	2.27E-05	0.148	0.2808	3.24E-02	0.07104	44
kegg_5.4	NA	NA	NA	NA	NA	NA	NA	NA	NA	0
kegg_5.5	NA	NA	NA	NA	NA	NA	NA	NA	NA	0
kegg_5.6	NA	NA	NA	NA	NA	NA	NA	NA	NA	0
kegg_5.7	NA	NA	NA	NA	NA	NA	NA	NA	NA	5
kegg_5.8	-0.231	7.09E-01	-0.0469	0.795	9.15E-03	0.147	0.7142	2.33E-02	0.22465	8
kegg_5.9	NA	NA	NA	NA	NA	NA	NA	NA	NA	0
kegg_5.10	NA	NA	NA	NA	NA	NA	NA	NA	NA	0

TableS5 Correlation between transcript and protein level changes for protein subcellular location.

**subcellular location**	**rho_afb1**	**pvalue_afb1**	**slope_afb1**	**rho_ota**	**pvalue_ota**	**slope_ota**	**rho_zen**	**pvalue_zen**	**slope_zen**
cytoplasm	0.320	0.000	0.098	0.598	0.000	0.158	0.236	0.000	0.087
cytoskeleton	0.262	0.025	0.101	0.650	0.000	0.196	0.381	0.002	0.108
ER_lumen	0.366	0.001	0.061	0.570	0.000	0.153	0.202	0.052	0.037
ER_membrane	0.481	0.001	0.188	0.673	0.000	0.196	0.332	0.021	0.083
golgi_lumen	0.879	0.025	1.513	0.100	0.436	0.039	0.601	0.142	0.351
golgi_membrane	0.450	0.007	0.163	0.241	0.104	0.077	0.611	0.000	0.275
lysosome	0.678	0.069	0.089	0.667	0.074	0.212	0.652	0.080	0.152
mito_membrane	0.207	0.132	0.090	0.723	0.000	0.163	0.246	0.091	0.084
mito_non_membrane	0.245	0.000	0.074	0.535	0.000	0.146	0.350	0.000	0.105
nuclear_membrane	0.537	0.003	0.226	0.561	0.002	0.212	0.404	0.023	0.176
nuclear_non_membrane	0.271	0.000	0.102	0.621	0.000	0.189	0.221	0.000	0.078
other_predicted_membrane	0.362	0.000	0.131	0.705	0.000	0.234	0.349	0.000	0.168
peroxisome	0.287	0.077	0.147	0.699	0.000	0.215	0.229	0.130	0.072
plasma_membrane	0.404	0.000	0.115	0.536	0.000	0.160	0.238	0.000	0.108
secreted_curated	0.838	0.000	0.190	0.741	0.001	0.188	0.650	0.004	0.192
secreted_highly_likely	0.428	0.000	0.133	0.605	0.000	0.161	0.538	0.000	0.172

Table S6. Correlation and linear regression slope between transcript and protein level changes for protein location in AFB_1, OTA and ZEN samples. We only present protein locations for data size (number of genes) higher than 10, correlation value higher than 0.3 with p value lower than 0.01. Slope higher than 0.2 is coded by red color, and lower than 0.15 with green color.

protein location	AFB1		OTA		ZEN		data size
	rho	slope	rho	slope	rho	slope	
**Secreted**	0.428	0.133	0.605	0.161	0.538	0.172	174
**Plasma membrane**	0.404	0.115	0.536	0.16			285
**Cytoplasm**	0.32	0.098	0.598	0.158			1492
**Golgi apparatus membrane**	0.45	0.163			0.611	**0.275**	29
**Mitochondria membrane**			0.723	0.163			31
**ER membrane**	0.481	0.188	0.673	0.196			38
**Nuclear membrane**	0.537	**0.226**	0.561	**0.212**			25
**Others membrane**	0.362	0.131	0.705	**0.234**	0.349	0.168	114
**Cytoskeleton**			0.65	0.196	0.381	0.108	57
**ER lumen**	0.366	0.061	0.57	0.153			66
**Peroxisome**			0.699	**0.215**			26
**Mito non-membrane**			0.535	0.146	0.35	0.105	390
